# Population genetic signatures of a climate change driven marine range extension

**DOI:** 10.1038/s41598-018-27351-y

**Published:** 2018-06-22

**Authors:** Jorge E. Ramos, Gretta T. Pecl, Natalie A. Moltschaniwskyj, Jayson M. Semmens, Carla A. Souza, Jan M. Strugnell

**Affiliations:** 1 Institute for Marine and Antarctic Studies, University of Tasmania, Hobart, TAS 7001 Australia; 2 Centre for Marine Socioecology, 20 Castray Esplanade, Hobart, TAS 7001 Australia; 3 Fisheries Research, Department of Primary Industries, Nelson Bay, NSW 2315 Australia; 4 Department of Ecology, Environment and Evolution, La Trobe University, Melbourne, VIC 3086 Australia; 5 Centre for Sustainable Tropical Fisheries and Aquaculture, James Cook University, Townsville, QLD 4811 Australia

## Abstract

Shifts in species distribution, or ‘range shifts’, are one of the most commonly documented responses to ocean warming, with important consequences for the function and structure of ecosystems, and for socio-economic activities. Understanding the genetic signatures of range shifts can help build our knowledge of the capacity of species to establish and persist in colonised areas. Here, seven microsatellite loci were used to examine the population connectivity, genetic structure and diversity of *Octopus tetricus*, which has extended its distribution several hundred kilometres polewards associated with the southwards extension of the warm East Australian Current along south-eastern Australia. The historical distribution and the range extension zones had significant genetic differences but levels of genetic diversity were comparable. The population in the range extension zone was sub-structured, contained relatively high levels of self-recruitment and was sourced by migrants from along the entire geographic distribution. Genetic bottlenecks and changes in population size were detected throughout the range extension axis. Persistent gene flow from throughout the historical zone and moderate genetic diversity may buffer the genetic bottlenecks and favour the range extension of *O. tetricus*. These characteristics may aid adaptation, establishment, and long-term persistence of the population in the range extension zone.

## Introduction

In response to the accelerated warming of the oceans, marine species can experience changes in their patterns of geographic distribution and abundance as they track their preferred temperatures^1,2^. Such range shifts occur in the form of range contractions, relocations, or extensions that have ecological and socio-economic impacts^3,4^, with already significant consequences on human well-being^5^. Most of our understanding of how genetic and evolutionary processes may relate to marine range shifts is based on terrestrial invasive or range shifting species^6–8^ and on theoretical studies^9–11^, with only a handful of empirical marine studies^12,13^. However, a range of different barriers influence the connectivity and genetic patterns of marine populations in comparison to terrestrial populations; these include water masses, currents, eddies, and coastlines, among other factors. As a consequence, the nature and magnitude of genetic patterns and evolutionary processes related to marine range shifts may differ from those that occur in the terrestrial realm. Therefore it is important to perform marine field-based studies that can increase our understanding of genetic patterns and evolutionary processes related to marine range shifts and help validate theoretical models.

Maintaining genetic diversity is essential if populations are to persist during range shifts associated with oceanic warming^12^. Selection, the reproductive exchange between individuals, dispersal capacity and the presence of barriers to dispersal, can all influence genetic diversity^6,14,15^ and survival at the trailing edge, at the centre of the distribution, and at the leading edge of range shifts^6,10^. Populations at the trailing edge may be negatively affected due to low genetic variability unless they receive genes from better adapted populations^6^. Populations at the centre of the distribution are likely to have more genetic variability due to gene flow from neighbouring populations that are better adapted to different conditions^6,16^. Genetic diversity at the leading edge of range shifts can increase provided there is gene flow from different areas of the distribution^6,16^, with maximum survival predicted to occur if the gene flow is from areas with similar conditions compared with the newly colonised areas^10,17^.

Genetic diversity can decrease due to genetic drift under scenarios of limited gene flow, genetic recombination and selection against poorly adapted genotypes^6^. Consecutive genetic bottlenecks or founder effects, where the size of the population is reduced or where only few individuals establish in new areas and subsequently become isolated, can result in genetic drift and inbreeding depression^18–20^. There are exceptions where invasive populations have overcome the negative impacts of the loss of genetic diversity via multiple introductions that aid the increase of genetic recombination, confer high phenotypic plasticity to the founder population, and strengthen its ability to respond to natural selection^7,21,22^. For instance, multiple introductions of individuals appeared to help sustain genetic diversity of the invasive long-spined sea urchin *Centrostephanus rodgersii* at the southern Tasman Sea off Australia’s east coast^12^. This area is warming up to four times faster than the global average^23^, partly caused by the strengthening of the warm East Australian Current (EAC) which flows polewards from the southern Coral Sea along the south-east coast of mainland Australia^24^. Over the past 60 years the EAC has strengthened and extended approximately 350 km further south towards the temperate east coast of Tasmania^25,26^.

**Figure 1:**
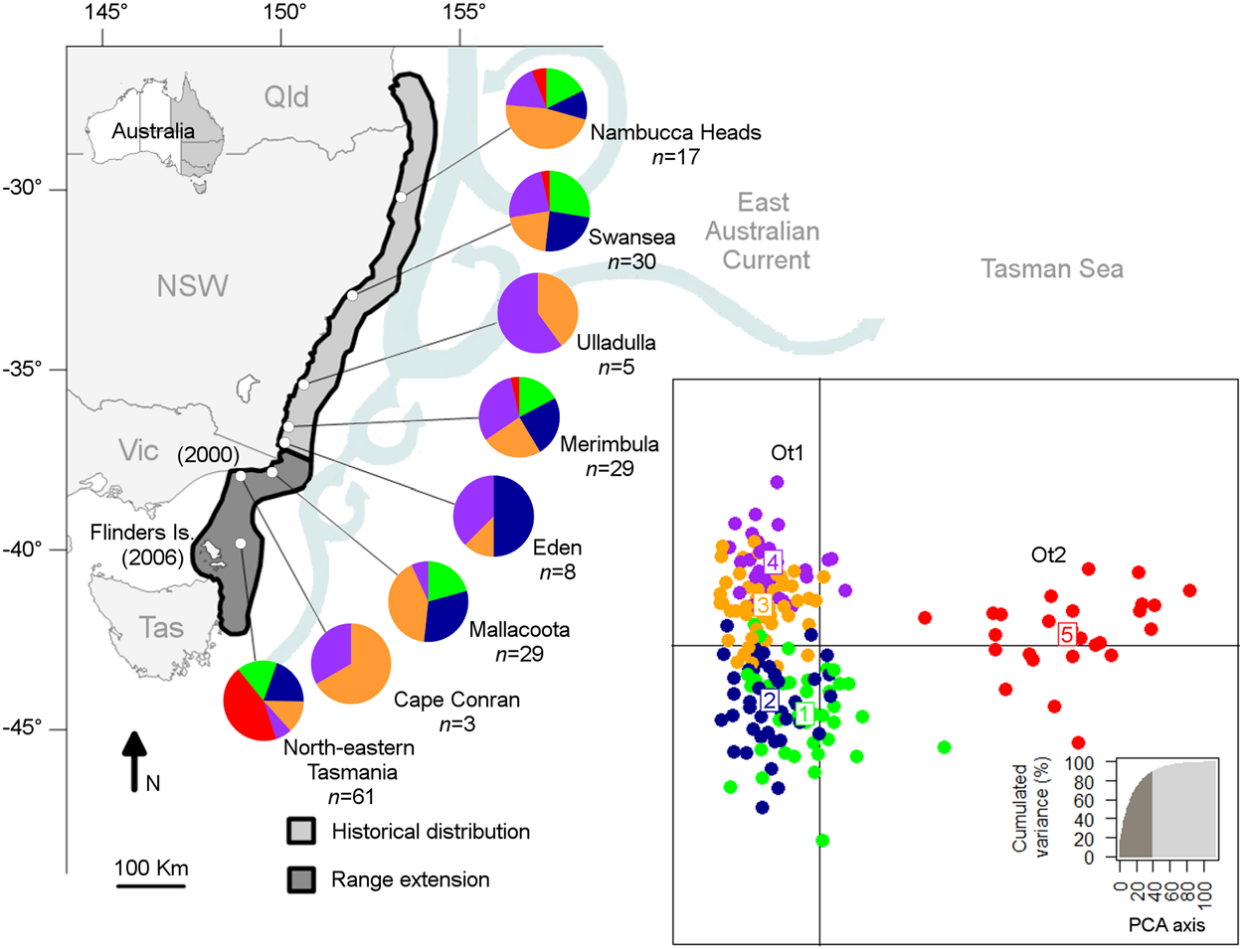
Collection sites for *Octopus tetricus* along eastern Australia, including historical and extension zones. Numbers in parentheses indicate the approximate years when *O. tetricus* individuals were detected for the first time off Victoria and off north-eastern Tasmania. The pie charts indicate the percentage of individuals from each site that correspond to each cluster assigned by colour (Cluster 1 – green; Cluster 2 – blue; Cluster 3 – orange; Cluster 4 – purple; Cluster 5 – red). The right panel is the graphic representation of the percentage contribution of individuals to clusters using Discriminant Analysis of Principal Components with the genetic structure being captured by the first three principal components. The Group Ot1 is comprised of individuals from all sites. The distinct Group Ot2 is predominately comprised of individuals from Tasmania (indicated in red). The map was modified from^30^.

The strengthening of the EAC has been associated with the transport of several dozen marine species during their planktonic larval stage, and their subsequent polewards range shift^27,28^. One of these range-shifting species is the gloomy or common Sydney octopus *Octopus tetricus* (Gould, 1852). This commercially important octopus has a short life span (~11 months)^29^, high reproductive capacity^30^ and a planktonic paralarval phase. The paralarval phase is likely to last 35–60 days as demonstrated for the closely related *O. vulgaris*^31,32^ under laboratory conditions^33,34^. The historical geographic range of *O. tetricus* is in shallow-waters from southern Queensland to southern New South Wales (NSW)^35–37^. *Octopus tetricus* was detected for the first time off Victoria around the year 2000 and off north-eastern Tasmania around 2006 (Fig. 1). Presence-absence records of *O. tetricus* along the east coast of Australia that support the polewards range extension hypothesis include systematic marine life censuses^36,38^, fisheries records^39^, and citizen science monitoring using scientist-verified and geo-referenced photographs^40^. Using a cost-effective and rapid screening assessment tool based on monitoring data, the range extension of *O. tetricus* was classified with a “high” level of confidence^41^. Negative impacts on the structure and function of the Tasmanian reef community are anticipated if the size of the *O. tetricus* population increases in Tasmanian waters^42^.

Examination of the genetic structure, level of connectivity and genetic diversity of range shifting populations will provide valuable information on the genetic signatures during range shifts and may allow forecasting of successful colonisations as a function of gene flow and genetic diversity^20^. Therefore, using *O. tetricus* as a model range extending marine species, this study aims: (1) to describe the population genetic structure of *O. tetricus* along eastern Australia, including extension areas; (2) to quantify the genetic diversity of the range extension area versus other population components; (3) to determine the level of gene flow between historical and range extension areas; (4) to identify the source populations that contribute to the range extension area; (5) to determine if there is evidence of genetic bottlenecks that may negatively affect the persistence of *O. tetricus* at the extended areas; and (6) to describe the recent evolutionary history of this range extending species.

## Results

A total of 182 individuals of the common Sydney octopus, *Octopus tetricus*, were genotyped for seven microsatellite loci (Table 1). Approximately 85% of pairs of loci (89 out of 105 tests over sites with ≥17 samples) were in linkage equilibrium (Supplementary Tables S1, S2). Exclusion of loci involved in linkage disequilibrium comparisons did not improve exploratory analyses. Moreover, Hardy-Weinberg equilibrium was met in 90% of each of the site-locus comparisons (Supplementary Tables S3, S4); therefore, all loci were included in further analyses. There was no evidence of large-allele dropout; stuttering was suggested for loci Ovul02 and Ovul16, as indicated by the shortage of heterozygote genotypes with alleles of one repeat unit difference. Moderate levels of null alleles (false homozygotes) were found in loci Ovul01 (10%), Ovul02 (14%), Ovul05 (≤19%), Ovul16 (≤13%), and at Tasmania (<14%); however, allele frequency bias was corrected. There were some differences in the results estimated from uncorrected data compared with results estimated from corrected data for null alleles. Therefore we present and discuss results based on corrected data where any bias is rectified. Results based on uncorrected data are presented in the Supplementary information online for comparative purposes only.

**Table 1:** Variability across seven polymorphic microsatellite loci in *Octopus tetricus* from the east coast of Australia.

Locus	Accession number	Repeat motif	Primer sequence (5′–3′)	Ta (°C)	Size range (bp)	N_A_	H_O_	H_E_
Ovul01	JN579690	(TG)_15_N(TG)_6_N(TG)_6_	AGATGAGGCAAAAGCAGAATA GAATGACTTCATAAAGCCACCT	65	252–263	7.8	0.680	0.749
Ovul02	JN579691	(GC)_4_N(AC)_31_	ACTGCCTGCCACTGTCTC ATTTGATTTACTCACATCGGGTT	65	251–302	24.6	0.847	0.956
Ovul05	JN579694	(AG)_4_N(GA)_6_N(TG)_5_N(AG)_5_AA(AG)_8_	GGAAGGAGAAGGACGAGAG CCTCCCACGAACACTCAT	65	235–263	7.2	0.328	0.427
Ovul08	JN579697	(AC)_5_N(CA)_10_N(AC)_8_N(TC)_8_N(TC)_4_	CCGTCAGATTATGCCAACAC GCGAGTGAAGGGGAAGTAGA	67	322–345	4.8	0.351	0.340
Ovul09	JN579698	(GT)_20_(GA)_18_N(GA)_4_	GGAAGGAATAAGAACAGAGAACG ATCTCTAATCTTCATTGCGTCTAA	62	367–397	14.2	0.863	0.894
Ovul14	JN579703	(GT)_4_GCT(TG)_31_N(GT)_5_N(TG)_4_	GGTGGGTGGCTGGTTTGACTACC CACTCAGGCAAATAGGGAAC	60	261–282	8.8	0.735	0.810
Ovul16	JN579705	(GT)_8_GCA(TG)_4_	AAGGGGCTGGTGACATTG CACTGGCATACTACATCAAACC	65	148–158	4.2	0.323	0.363

Ta – annealing temperature; N_A_ – number of alleles; H_O_ – observed heterozygosity; H_E_ – expected heterozygosity. Microsatellite loci modified from^59^.

### Population sub-structuring

Discriminant Analysis of Principal Components (DAPC) suggested five clusters (Table 2; Supplementary Fig. S5 and Table S6): (1) green, (2) blue, (3) orange, (4) purple, and (5) red, with 40 Principal Components explaining >80% of cumulated variance. Clusters one to four were largely overlapped, i.e. comprised of a mixture of individuals from along the entire distribution and there was no clear differentiation between these clusters. Therefore, clusters one to four were pooled in the common Group Ot1 (Fig. 1, right panel). Cluster five (red) was separated from the rest of the clusters and was mostly comprised of individuals from Tasmania in the range extension zone (n = 27), but also contained a few individuals from Nambucca Heads (n = 1), Swansea (n = 1) and Merimbula (n = 1) in the historical distribution zone; this distinct group was termed Ot2 (Fig. 1, right panel). Tasmania (n = 61) was thus sub-structured with 56% (n = 34) individuals that belonged to the common Group Ot1, and with 44% (n = 27) individuals that belonged to the genetically distinct Group Ot2. DAPC analyses carried out on a range of subsets of the dataset, i.e. (a) at all sites within mainland Australia only, (b) at the historical distribution zone only, (c) at the range extension zone only, (d) Tasmania only, and (e) not including the distinct Group Ot2, showed similar population structure. The software Structure suggested only four clusters (Table 2; Supplementary Fig. S5 and Table S6), for which individual allocation was not as obvious as for DAPC. Most individuals assigned to clusters one to four in DAPC were allocated to clusters one to three by Structure, which comprise the common Group Ot1. Most individuals assigned to cluster five in DAPC were allocated to cluster four by Structure, which comprise the distinct Group Ot2 (Table 2; Supplementary Table S6 and Fig. S7).

**Table 2:** Percentage (%) contribution of *Octopus tetricus* individuals from the east coast of Australia to assigned clusters estimated in DAPC and Structure.

Site	DAPC					Structure			
	1	2	3	4	5	1	2	3	4
Nambucca Heads	18	12	47	18	6	18	59	24	0
Swansea	28	24	21	24	3	37	40	17	7
Ulladulla	0	0	40	60	0	0	100	0	0
Merimbula	17	24	24	31	3	34	38	21	7
Eden	0	50	13	38	0	38	38	25	0
Mallacoota	21	31	41	7	0	31	34	31	3
Cape Conran	0	0	67	33	0	0	67	33	0
Tasmania	16	20	13	7	44	31	10	16	43

**Figure 2:**
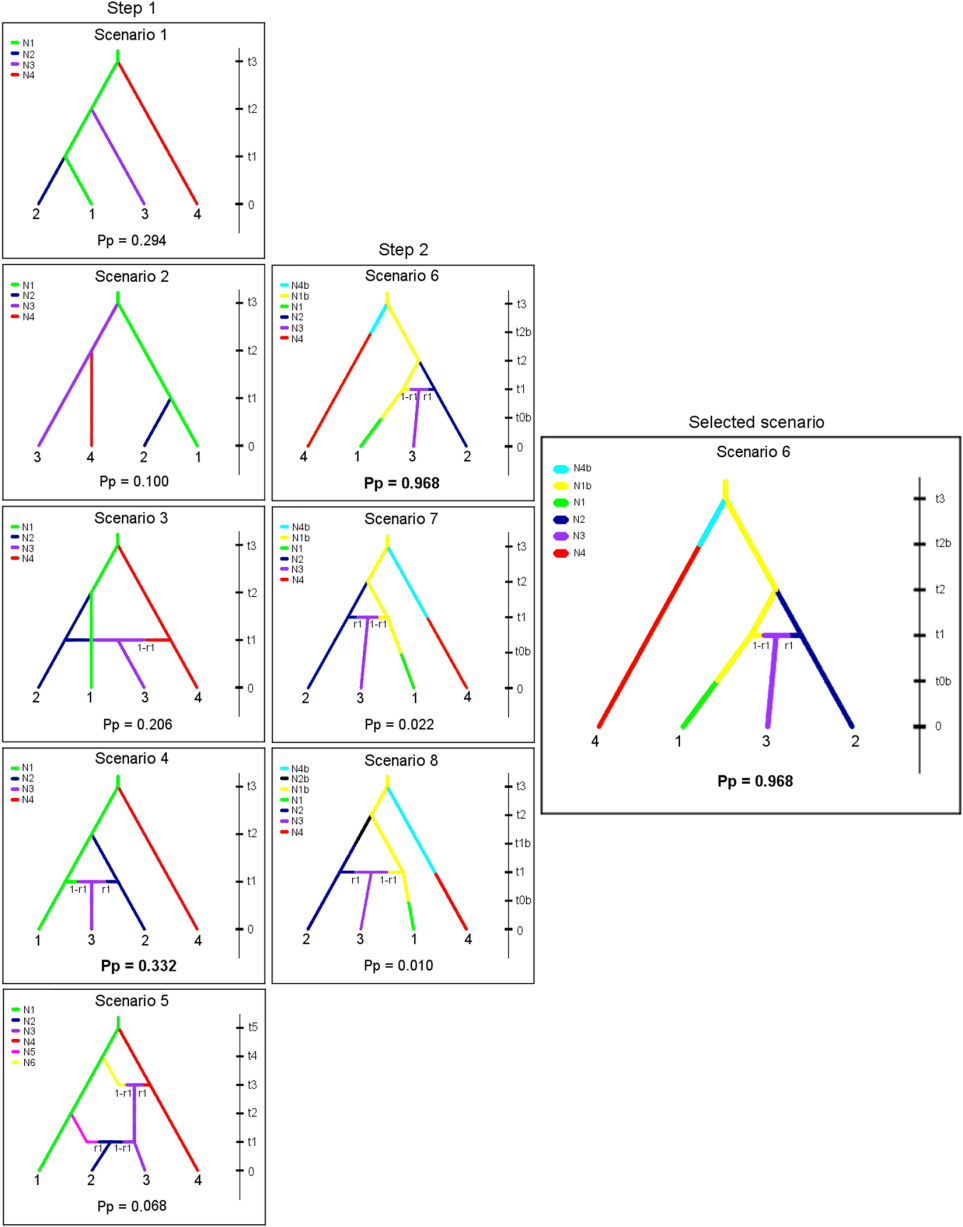
Hierarchical Approximate Bayesian Computation analysis of population history scenarios of the range extending *Octopus tetricus* along the east coast of Australia. In step 1, the scenario 4 was selected as the most likely population topology. In step 2, scenario 6 was selected as the most accurate model of demographic history for our data. Sub-group 1 – Nambucca Heads, Swansea, and Merimbula; Sub-group 2 – Mallacoota; Sub-group 3 – Ot1 Tasmania. The common Group Ot1 is comprised of the sub-groups 1 to 3. Sub-group 4 – distinct Group Ot2 predominately comprised of individuals from Tasmania. Ulladulla, Eden and Cape Conran were not included in this analysis due to their small sample sizes (n < 17). The colours represent different sub-groups and changes in the size of their population. Times are not to scale. Selected scenarios at each step are indicated by posterior probabilities (Pp) in bold font.

DAPC – Discriminant Analysis of Principal Components; Cluster 1 – green; Cluster 2 – blue; Cluster 3 – orange; Cluster 4 – purple, and Cluster 5 – red in Figs 1 and 2 and in Supplementary Fig. S7. Nambucca Heads (n = 17); Swansea (n = 30); Ulladulla (n = 5); Merimbula (n = 29); Eden (n = 8); Mallacoota (n = 29); Cape Conran (n = 3); Tasmania (n = 61).

### Phylogenetic analysis

Phylogenetic analysis of the mitochondrial gene Cytochrome Oxidase subunit I (COI) demonstrated that individuals of the groups Ot1 and Ot2 correspond to *O. tetricus*. None of the individuals analysed were assigned to any other species that are closely related to *O. tetricus*, e.g. *O*. cf *tetricus* or *O. vulgaris* (Supplementary Fig. S8).

### Genetic diversity

Allelic richness was moderate with more than seven alleles at each site; the number of private alleles and heterozygosity in the range extension zone were higher than for the historical distribution zone, and inbreeding coefficients were not significant (Table 3; Supplementary Tables S3, S4). Overall, moderate levels of genetic diversity were observed across sites and loci, and genetic diversity was similar at the range extension compared with the historical distribution zone (Table 3).

**Table 3:** Descriptive statistics for *Octopus tetricus* along the east coast of Australia. n – number of individuals genotyped; N_A_ – number of alleles; N_PA_ – number of private alleles; H_O_ – observed heterozygosity; H_E_ – expected heterozygosity; A_R_ – allelic richness (rarefied to 17 samples); F_IS_ – inbreeding coefficient (F_IS_ values were not significant at P < 0.05); P value – significance for Hardy-Weinberg equilibrium (P < 0.05).

	Nambucca Heads	Swansea	Merimbula	Mallacoota	Tasmania	Group Ot1	Group Ot2
n	17	30	29	29	61	152	30
N_A_	7.714	10.571	10.429	10.286	13.857	16.714	11.000
N_PA_	0.429	0.857	0.714	0.714	2.000	7.286	1.714
H_O_	0.560	0.632	0.650	0.645	0.705	0.635	0.712
H_E_	0.571	0.657	0.659	0.644	0.714	0.638	0.691
A_R_	7.563	8.297	8.349	8.261	8.613	9.019	9.899
F_IS_	0.005	0.035	0.025	−0.011	−0.006	<0.001	−0.070
P value	0.344	0.208	0.528	0.631	0.551	0.267	0.678

The Group Ot1 is comprised of individuals from all sites, including Ulladulla, Eden and Cape Conran. The distinct Group Ot2 is predominately comprised of individuals from Tasmania (indicated in red in Figs 1 and 2 and in Supplementary Fig. S7). Descriptive statistics were not presented for Ulladulla, Eden and Cape Conran due to their small sample sizes (n < 17).

### Genetic connectivity and differentiation

F_ST_ estimations provided further detail of the population structure and complemented DAPC and Structure results. There was no significant differentiation between Nambucca Heads, Swansea, and Merimbula (Table 4; Supplementary Table S9). Low but significant F_ST_ values indicated that Mallacoota was different from all other sites, except from Swansea. Tasmania was different from all sites along mainland Australia (Table 4; Supplementary Table S9). Exploratory analysis of population differentiation indicated that Ot2 Tasmanian individuals were differentiated from all other sites including the Ot1 Tasmanian individuals (F_ST_ = 0.147, P < 0.001). Thus, from the F_ST_ estimations we inferred that the common Group Ot1 was comprised of sub-group 1 (Nambucca Heads, Swansea, and Merimbula), sub-group 2 (Mallacoota), and sub-group 3 (Ot1 Tasmanian individuals). The distinct Group Ot2 was comprised of the sub-group 4 (Ot2 Tasmanian individuals). The F_ST_ analysis at the Group level confirmed that the common Group Ot1 was significantly different from the Group Ot2 (F_ST_ = 0.157, P < 0.001). The Mantel test indicated that the level of genetic differentiation among collection sites was not significantly correlated to geographic distance (P = 0.258) or year of collection (P = 0.096). Accordingly, the Spearman’s correlation analysis suggested that only two loci were significantly correlated to year of collection (Ovul01, r_s_ = 0.231, P = 0.002; Ovul08, r_s_ = 0.148, P = 0.047). Two loci were associated to depth of sampling (Ovul01, r_s_ = −0.230, P = 0.002; Ovul05, r_s_ = −0.176, P = 0.018) (Supplementary Table S10). The AMOVA detected significant percentages of genetic variation among collection sites (3.3%, d.f. = 7, SS = 30.950, VC = 0.062, P < 0.001).

**Table 4:** F_ST_ among collection sites for *Octopus tetricus* along the east coast of Australia.

Site	Nambucca Heads	Swansea	Merimbula	Mallacoota	Tasmania
Nambucca Heads	—	0.4314	0.5322	**0.0208**	**<0.001**
Swansea	0.0009	—	0.8643	0.0606	**<0.001**
Merimbula	−0.0008	−0.0046	—	**0.0128**	**<0.001**
Mallacoota	**0.0160**	0.0083	**0.0128**	—	**<0.001**
Tasmania	**0.0463**	**0.0302**	**0.0322**	**0.0449**	—

F_ST_ values are indicated below the diagonal. P values are indicated above the diagonal. Bold indicates significant values at P < 0.05. F_ST_ was not estimated for Ulladulla, Eden and Cape Conran due to their small sample sizes (n < 17).

### Migration and self-recruitment

Migration rates were asymmetric between sites, with higher migration rates from Merimbula to the other sites (Table 5; Supplementary Table S11). Migration rates were asymmetric between the common Group Ot1 and the distinct Group Ot2, with greater migration rates from the common Group Ot1 towards the distinct Group Ot2 (0.095 ± 0.029 SD), than from the distinct Group Ot2 towards the common Group Ot1 (0.025 ± 0.008 SD). Merimbula had the highest level of self-recruitment (0.914 ± 0.024) (Table 5; Supplementary Table S11). Both groups Ot1 and Ot2 had high levels of self-recruitment with slightly higher self-recruitment in Ot1 than in Ot2 (0.975 ± 0.008 and 0.905 ± 0.029, respectively).

**Table 5:** Inferred (posterior mean) migration rates of *Octopus tetricus* between collection sites along the east coast of Australia.

Site	Nambucca Heads	Swansea	Ulladulla	Merimbula	Eden	Mallacoota	Cape Conran	Tasmania
Nambucca Heads	*0.680* (*0.013)*	0.014 (0.013)	0.014 (0.013)	0.219 (0.032)	0.014 (0.013)	0.014 (0.013)	0.014 (0.013)	0.032 (0.021)
Swansea	0.009 (0.009)	*0.676* (*0.009)*	0.009 (0.009)	0.249 (0.025)	0.009 (0.009)	0.009 (0.009)	0.009 (0.009)	0.032 (0.018)
Ulladulla	0.025 (0.024)	0.026 (0.024)	*0.694* (*0.025)*	0.153 (0.044)	0.026 (0.024)	0.026 (0.024)	0.025 (0.023)	0.026 (0.024)
Merimbula	0.009 (0.009)	0.009 (0.009)	0.009 (0.009)	*0.914* (*0.024)*	0.009 (0.009)	0.009 (0.009)	0.009 (0.009)	0.033 (0.017)
Eden	0.021 (0.020)	0.021 (0.020)	0.021 (0.020)	0.186 (0.040)	*0.687* (*0.020)*	0.021 (0.020)	0.021 (0.020)	0.021 (0.020)
Mallacoota	0.009 (0.008)	0.009 (0.009)	0.009 (0.009)	0.268 (0.023)	0.009 (0.009)	*0.676* (*0.009)*	0.009 (0.009)	0.011 (0.010)
Cape Conran	0.030 (0.028)	0.030 (0.028)	0.031 (0.028)	0.120 (0.046)	0.030 (0.028)	0.030 (0.028)	*0.699* (*0.029)*	0.030 (0.028)
Tasmania	0.005 (0.005)	0.005 (0.005)	0.005 (0.005)	0.192 (0.021)	0.005 (0.005)	0.005 (0.005)	0.005 (0.005)	*0.778* (*0.020)*

Italicised values indicate self-recruitment. Values in parentheses are the standard deviations of the marginal posterior distribution for each estimate. Left column indicates where migrants travelled to; top row indicates where migrants originated from.

### Genetic bottleneck analysis

Heterozygosity excess as an indication of genetic bottleneck was detected under the Stepwise Mutation Model (SMM) at all sites, except for the sign test for Nambucca Heads. Under the Two-Phase Mutation Model (TPM), heterozygosity excess was detected at Tasmania. Genetic bottleneck effect was also detected at the distinct Group Ot2 and at the Group Ot1 under the SMM and the TPM. Tests under the Infinite Allele Model (IAM) and the mode-shift test did not detect genetic bottleneck effects at any site (Table 6; Supplementary Table S12).

**Table 6:** Heterozygosity excess tests results to detect genetic bottleneck on *Octopus tetricus* along the east coast of Australia.

Site/Group	SMM		TPM		IAM		
	i	ii	i	ii	i	ii	iii
Nambucca Heads	0.116	**0.039**	0.116	0.375	0.365	0.688	L-shaped
Swansea	**0.021**	**0.023**	0.282	0.297	0.596	0.813	L-shaped
Merimbula	**0.021**	**0.023**	0.095	0.109	0.593	1.000	L-shaped
Mallacoota	**0.020**	**0.023**	0.098	0.078	0.290	0.688	L-shaped
Tasmania	**0.020**	**0.016**	**0.021**	**0.039**	0.577	1.000	L-shaped
Group Ot1	**0.001**	**0.008**	**0.021**	**0.016**	0.288	0.578	L-shaped
Group Ot2	**0.002**	**0.008**	**0.019**	**0.016**	0.582	1.000	L-shaped

The Group Ot1 is comprised of individuals from all sites. The distinct Group Ot2 is predominately comprised of individuals from Tasmania (indicated in red in Figs 1 and 2 and in Supplementary Fig. S7). Values for sites with ≥17 samples are presented only. SMM – Stepwise Mutation Model; TPM – Two-Phase Mutation Model. IAM – Infinite Allele Model; i – “Sign test”; ii – “2-tailed Wilcoxon sign rank test”; iii – “Mode-shift test”. L-shaped – no bottleneck effect detected. Significance at P < 0.05, with significant bottleneck effect indicated in bold.

### Population history

In the first step, scenario 4 was selected as the best population topology (Pp = 0.332) (Fig. 2, colour coding in Fig. 2 relates to the clusters identified in the population structure analysis; Supplementary Tables S13, S16). In the second step, scenario 6 had the highest cumulative posterior probability (P = 0.968) and was selected as the most accurate model of demographic history for our data. In this scenario, sub-group 4 (distinct Group Ot2) diverged from sub-group 1 (Nambucca Heads, Swansea, and Merimbula) relatively early in the history of the population (t3, 95% CI). One genetic bottleneck occurred to sub-group 4 relatively early in time (t2b), with a subsequent increase in the size of its population, from *Ne* = 286 (N4b) to *Ne* = 7,930 (N4) at t2 (95% CI). Sub-group 1 experienced a recent genetic bottleneck (t0b), with a posterior decrease in the size of its population from *Ne* = 8,570 (N1b) to *Ne* = 7,220 (N1). Sub-group 2 (Mallacoota; *Ne* = 4,200) diverged from sub-group 1 at t2 (95% CI), whereas sub-group 3 (Ot1 Tasmania; *Ne* = 5,060) diverged from the admixture of sub-groups 1 and 2 at t1 (95% CI) (Fig. 2; Supplementary Tables S17–S20). Posterior estimations of all parameters for the selected scenario were consistent when an alternative set of priors was used.

## Discussion

The key findings of this study are that genetic differentiation was detected between sites present within the historical distribution and the range extension zones, and also within the range extension zone. The population was sub-structured in one common group along eastern Australia and Tasmania (Group Ot1), and a distinct group mainly comprised of individuals from Tasmania (Group Ot2). Migration rates were asymmetrical with greater migration occurring from the historical distribution zone towards sites at the range extension zone. The genetic diversity at the range extension zone was comparable to that detected at the historical distribution area. Genetic bottleneck effects were detected at sites within the historical distribution and range extension zones. Population history simulations suggest that the distinct group diverged from the common group relatively early in the history of the population.

Systematic marine life censuses^36,38^, fisheries records^39^, and citizen science monitoring^40^ suggest that *O. tetricus* has recently extended its distribution polewards into Tasmanian waters. Moreover, this range extension was classified with “high” level of confidence^41^ and we detected genetic signatures that are characteristic of range shifts^9^. The presence of the common Group Ot1 throughout the historical distribution and the range extension zones is supported by the detected gene flow between all sites and by shared haplotypes in *O. tetricus* between NSW and Tasmania using mitochondrial DNA^32^. In contrast, Ot2 individuals were common in Tasmanian waters but uncommon along the east coast of mainland Australia. This finding is consistent with the increase in size of the Group Ot2, detected by the Approximate Bayesian Computation analysis (ABC), probably from a genotype that is relatively uncommon in the historical distribution zone. The differentiation and expansion in size of the Group Ot2 was likely favored by high self-recruitment, rapid population turnover and high reproductive capacity^29,30^. These characteristics provide species with high evolutionary capacity^43^ and may facilitate long-term persistence in range extension zones^44^. However, the range extension of *O. tetricus* appears to be rather complex. A genetic break was detected in Tasmanian waters between the Group Ot2 and Ot1 Tasmanian individuals. This genetic break is beyond the scope of this study and requires further investigation to understand the mechanisms that may cause limited mixing of both groups despite coexisting in the same area, e.g. differential selection, non-random mating or short-term departures from local panmixia. In addition, the ABC simulations estimated that the Group Ot1 appeared in Tasmanian waters more recently than the Group Ot2, which diverged early in the history of the population. These signatures suggest that *O. tetricus* historically diverged between Tasmania and mainland Australia, and secondary contact occurred due to the recent expansion of the mainland population polewards towards the south.

Following theoretical expectations^10^, the ABC analysis suggested a decrease in size of the population at the equatorward sites. A relatively high level of heterozygosity suggests that Tasmania is within the leading edge of the range extension; this finding is consistent with the genetic pattern observed at the leading edge of range shifts of Quaternary trees in response to climate change^6^. The range shift of *O. tetricus* thus appears to consist in the polewards relocation of the population, with characteristics of recent and rapid demographic expansions^9^. Similarly, populations of *C. rodgersii* undergoing rapid range expansions had high levels of genetic diversity and heterozygosity at the leading edge of the range^12^. Constant gene flow from along the entire distribution aids sustained genetic diversity in extension areas^21^, which can confer phenotypic plasticity to founder individuals and the ability to respond to natural selection^7,22^. Accordingly, discrete morphological differences were found in male *O. tetricus* between the east coast of mainland Australia and Tasmania^32^.

The population structure and connectivity of *O. tetricus* is expected to be shaped by the dynamics of the EAC^24–26^, which is likely the main driver of larval transport along the east coast of Australia^27,28^. With an assumed planktonic duration of 35–60 days, as estimated for *O. vulgaris*^33,34^, paralarvae of *O. tetricus* can be transported the linear distance between Nambucca Heads and Tasmanian waters (~1,150 km) at an average seawater flow of 55 cm·s^−1 ^^24^. Our findings are supported by the connectivity detected between the east coast of mainland Australia and Tasmania^32^. However, the use of Euclidean distances may be a poor predictor of gene flow^45^ because the effect of the coastline creates circuitous, turbulent, and nonlinear flow, which in addition to eddies present along the east coast of Australia may result in patchy larval dispersal^46–48^.

Collection sites of this study are exposed to a gradient of temperatures from NSW (annual average SST 20.2 ± 0.2 SE °C at −32° 31′ 15′′ and 152° 28′ 45′′) to north-eastern Tamania (annual average SST 15.2 ± 0.2 SE °C at −39° 33′ 45′′ and 148° 13′ 45′′). Such a gradient of environmental conditions also may have aided in shaping the population structure of *O. tetricus*^49^. Constant gene flow from along different environmental conditions within the historical distribution zone, and subsequent interbreeding, can assist in maintaining genetic diversity, create new gene complexes^21,50,51^, and buffer against any new set of environmental stressors in the range extension zone^8,52^. Moreover, Merimbula was the main source of migrants towards the range extension zone. This site is located at the historical polewards edge of *O. tetricus* range and shares more similar environmental conditions (i.e. temperature) with the range extension zone than other sites further equatorward. Such similarities may further facilitate the capacity of founder individuals to respond to selective pressure at the range extension zone^17^. Maintenance of genetic diversity by sufficient gene flow is therefore likely to contribute to the establishment, early success and persistence of *O. tetricus* in the extension zone^16,21^.

The polewards dispersion of larvae is projected to intensify gradually over the next decades along the east coast of Australia^53^, likely facilitating the population connectivity between historical and range extension zones. However, the accelerated warming along the east coast of Australia is anticipated to shorten the paralarval phase of *O. tetricus*. A shorter paralarval phase may reduce dispersal capacity, population connectivity^54,55^, and recovery of genetic diversity in the range extension area. Effects of ocean warming on reproductive seasons, frequency of paralarvae releases, swimming capabilities, paralarvae settlement windows, habitat suitability, and paralarvae mortality may also influence the connectivity of populations^46^. Therefore ‘seascape genetics’ studies must include oceanographic, life history, ecological and physiological data, in the context of ocean warming and changes in oceanic circulation into the examination of the population connectivity. This approach may provide better understanding of the structure, connectivity, genetic diversity, and capacity of populations to prevail in the new sections of their geographic distribution^56^. *Octopus tetricus* has a life-span of 11 months^29^ and allele frequencies could change between years of collection. To strengthen the robustness of our conclusions, future research should include larger samples sizes and from consecutive years within sites to assess stability of allele frequencies through time.

This study suggests that the range shift of *O. tetricus* is characterized by a genetically diverse range extension zone. Constant gene flow from a diversity of source areas along the entire distribution may promote relatively high genetic diversity and counteract genetic bottleneck effects at extension areas. Therefore, the genetic signatures examined in this study suggest that *O. tetricus* is well placed to be able to persist in its range extension zone provided that no demographic or environmental effect negatively affects the population.

## Methods

This research was conducted under the permits no. A11591 and A13740 approved by the University of Tasmania Animal Ethics Committee and in accordance with the ‘Australian code of practice for the care and use of animals for scientific purposes’^57^.

### Specimen collection

Octopus were collected from inshore waters along the NSW coast at Nambucca Heads (n = 17; −30° 38′ 46′′, 153° 0′ 12′′) and Swansea (n = 30; −33° 5′ 9′′, 151° 38′ 20′′) during February 2014; and at Ulladulla (n = 5; −35° 19′ 20′′, 150° 31′ 29′′), Merimbula (n = 29; −36° 53′ 42′′, 149° 54′ 25′′), and Eden (n = 8; −37° 4′ 18′′, 149° 54′ 33′′) during May 2013. Sites along the Victorian coastline were Mallacoota (n = 29; −37° 33′ 22′′, 149° 45′ 36′′) and Cape Conran (n = 3; −37° 48′ 49′′, 148° 43′ 37′′) with samples collected during May 2013. Samples also were collected off north-eastern Tasmania (n = 61; −39° 43′ 36′′, 148° 27′ 17′′) during April, September and December 2011. The centre of the known historical distribution included Nambucca Heads, Swansea, and Ulladulla; sites at the polewards edge of the historical distribution were Merimbula and Eden, whereas sites at the range extension zone were Mallacoota, Cape Conran, and Tasmania (Fig. 1). Octopus from Tasmania were collected on board of the *FV Farquharson* using black plastic shelter pots (0.3-m long × 0.1-m high × 0.1-m wide) laid on the seafloor at depths of 35–46 m. Octopus were euthanized by commercial fishers and immediately put in watery ice in an insulated container. A tissue sample was taken from the arm of every specimen and octopus carcasses were returned to the fishers. Octopus from Ulladulla were collected during diving activities whereas specimens from all other sites along the coast of mainland Australia were collected by hand while snorkelling at depths of 1–3 m. These animals were anesthetized by immersion in a 2% MgCl_2_ solution, a tissue sample was taken and octopus were released after recovery. All tissue samples were fixed in 95% ethanol.

### DNA extraction, PCR amplification and genotyping

DNA was extracted using the high salt method^58^ from a total of 182 animals. Seven microsatellite primers (Table 1) identified for *O. vulgaris*^59^ were amplified in *O. tetricus* and found to be polymorphic. The microsatellites loci revealed a moderate level of heterozygosity (Table 3) with two loci exhibiting more than 10 alleles at each site (Supplementary Tables S3, S4). Microsatellite loci were assigned unique fluorophores (FAM, VIC, NED, PET)^60^ to enable fluorescent tagging of PCR products.

PCR reactions were performed^59^, with modifications to the annealing temperature (Ta) (Table 1). Each PCR contained 4.725 µL of double distilled H_2_0, 6.25 µL of MyTaq Redmix (Bioline), 0.075 µL of 10 mM forward primer, 0.25 µL of 10 mM reverse primer, 0.20 µL of 5pmol/µL fluorophore labelled primer, and 1 µL (18–37 ng) of DNA. PCR conditions were modified slightly to optimize PCR products for some samples, such that 1 µL of 25 mM MgCl_2_ (Promega) was added in place of water. The number of cycles was reduced from 35 to 30, and the final extension was reduced from 5 to 3 min. Capillary separation of PCR products was performed by the Australian Genome Research Facility Ltd (AGRF). Genotypes were scored by eye using Geneious Pro v. 5.6.4^61^. PCRs were repeated up to three more times for individuals with unclear or missing single-locus genotypes before being categorized as missing data and scored as 000000 (n = 25).

### Genetic polymorphism

Micro-Checker v. 2.2.3^62^ was used to search for evidence of allele dropout and stuttering for each locus. High frequencies of null alleles are commonly observed in marine invertebrate species including molluscs^63–65^. The presence of null alleles can lead to overestimation of F_ST_ in cases of low levels of gene flow and significant population differentiation. Therefore, following the Expectation Maximization algorithm^66^, FreeNA^67^ was used to estimate null allele frequencies detected as false homozygotes. Null alleles were then corrected by re-naming them as 999 (e.g. a false homozygote with allele size 257257 was corrected to 257999). This method greatly reduces the bias of F_ST_ caused regardless of the frequency of null alleles, the level of gene flow, and the number of loci^67^.

### Population sub-structuring

The DAPC^68^ was used with the package ‘adegenet’^69^ in RStudio v. 0.99.435^70^ to test separately for population structure along the east coast of Australia and on a range of subsets of the dataset, i.e. (a) at all sites within mainland Australia, (b) at the historical distribution only, (c) at the range extension zone only, (d) Tasmania only, and (e) not including the distinct Group Ot2. The admixture and correlated allele frequencies models were also implemented in Structure v. 2.3.4^71^ to examine population structure. The number of clusters (K) explored was 1–10, with 10 independent runs of 500,000 Monte Carlo Markov Chain (MCMC) replicates and a burnin length of 50,000. The Evanno method^72^ implemented within Structure Harvester v. 0.6.93^73^ was used to evaluate the results, which were graphically displayed using Distruct v. 1.1^74^.

### Phylogenetic analysis

A phylogenetic analysis was performed to corroborate that individuals of the different groups detected from population structure analyses were in fact *O. tetricus* and not representative of a cryptic species (Supplementary methods online: ‘Phylogenetic analysis’).

### Genetic diversity

Descriptive statistics were estimated for each collection site with ≥17 samples, as well as for the groups inferred from the population structure analyses. Arlequin v. 3.5.1.3^75^ was used to test for genotypic linkage disequilibrium among loci within sites (20,000 iterations; P < 0.05), and for departures from Hardy–Weinberg equilibrium using the score test for heterozygote deficiency with level of significance determined by the Markov chain method (1,000,000 iterations). Arlequin v. 3.5.1.3^75^ was also used to estimate the number of alleles (N_A_), observed (H_O_) and expected (H_E_) heterozygosity and inbreeding coefficient (F_IS_). Allelic richness (A_R_) was rarefied to 17 samples using FSTAT^76^. The number of private alleles (N_PA_) was calculated using Convert v. 1.31^77^.

### Genetic connectivity and differentiation

Pairwise F_ST_ was calculated using Arlequin v. 3.5.1.3^75^ between all sites with ≥17 samples. A Mantel matrix correlation test was performed in Genepop web v. 4.2^78,79^ to examine if genetic differentiation (F_ST_) was explained by isolation by geographical distance (km) between collection sites (excluding Ulladulla, Eden, and Cape Conran with <17 samples). Isolation by year of collection was also tested for between collection sites, i.e. 2011 (Tasmania), 2013 (Mallacoota and Merimbula), and 2014 (Swansea and Nambucca Heads). A Spearman’s correlation analysis also was carried out to discount any effect of the sampling scheme by testing for an association between loci and year and depth of collection. In addition, AMOVA implemented in Arlequin v. 3.5.1.3^75^ was used to test for partitioning of genetic variation among collection sites.

### Migration and self-recruitment

BayesAss v. 3.0.1^80^ was implemented to assess admixture^81^. Migration rates (Δm), allele frequencies (Δa), and inbreeding coefficients (Δf) were given a mixing parameter value of 1 each for the analysis at the collection sites level, and a mixing parameter value of 0.2 each for the analysis at the groups level. We used 50,000,000 iterations, a 5,000,000 burnin length, and a 1000 interval between samples. Trace output convergence was assessed using Tracer v. 1.6^82^. Recent migration rates (i.e. within the recent 2–3 generations) were then estimated between collection sites, as well as between groups inferred from the population structure analyses.

### Bottleneck analysis

Heterozygosity excess is a genetic signature of genetic bottlenecks because alleles are usually lost faster than heterozygosity during a bottleneck as the mutation-drift equilibrium is lost^83^. Heterozygosity excess was tested with the software Bottleneck v. 1.2.02^84^, using 10,000 iterations for each site with ≥17 samples, and for the groups inferred from the population structure analyses. The tests used were: (i) The “sign test”^83^, (ii) the 2-tailed “Wilcoxon sign rank test”^85^, and (iii) the “mode-shift test”^86^. These tests allow the detection of the heterozygosity excess due to the faster loss of alleles at low-frequency class (<0.1) than alleles in 1 or more intermediate allele frequency classes^85,86^. For instance, the mode-shift test detects the L-shaped distribution of allele proportions observed at mutation-drift equilibrium, and bottlenecked populations that do not present the L-shaped distribution^86^. Microsatellite loci may tend to evolve under a model more similar to the SMM^87^ than to the IAM^83,85,87,88^. However, the SMM, the IAM, and a model in between both models, the TPM (using default settings)^89^ were examined. Examination of the three mutation models allowed greater caution regarding the detection of genetic bottlenecks.

### Population history

To reconstruct the history of divergence, migration and admixture events between the sub-groups identified based on the significant levels of genetic differentiation (F_ST_), datasets of historical and/or demographic events were simulated under a total of eight different scenarios using a coalescent-based ABC algorithm implemented in DIYABC v. 2.1^90^. The ABC approach relies on gene genealogy, demographic history and ancestral polymorphisms to explain evolutionary causes of present-day genetic variation, and thus can be used to discriminate among putative evolutionary scenarios. This approach is commonly used to distinguish the effect of contemporary recurrent gene flow from the effects of historical connectivity^91^. The ABC algorithm compares summary statistics of simulated datasets for each historical scenario and/or demographic events with summary statistics of the observed data. The posterior probability and distribution of parameters of each scenario are estimated and alternative scenarios can be compared.

The eight scenarios were examined in two successive steps. In the first step, five scenarios were examined: Scenarios 1 and 2 assumed successive independent divergence suggesting historical diversification; scenarios 3 and 4 assumed a single admixture event, and scenario 5 assumed two successive admixture events. We chose the scenario with the highest posterior probability (best fitted topology) from the first step. In the second step, three scenarios based on the best fitted topology selected in the first step were examined to refine changes in effective population size. These scenarios assumed a common origin of the sub-groups with successive divergence to test the hypothesis of recent colonization due to range extension. Thus, in the second step scenarios 6 and 7 considered changes in the effective number of founder specimens after bottlenecks and in the effective population size of cluster 4 at different generation times. Scenario 8 also considered changes in the effective number of founder specimens after bottlenecks and in the effective population size of other clusters (e.g. cluster 2).

To perform the ABC analysis a reference table with 3,000,000 coalescent-simulated data sets for each scenario was built, from which summary statistics parameter values were used in prior distributions (Supplementary Tables S13 and S17). Posterior probabilities were computed for each scenario applying linear discriminant analysis on summary statistics to find the best-supported scenario based on the direct approach estimates (500 selected data sets). The level of confidence in the chosen scenario was evaluated by estimating the type I and type II error rates based on simulated datasets. The marginal posterior distribution of each parameter was estimated based on the best model. The goodness of fit of the model-posterior parameter distribution combination was evaluated with the data. The scenario with the highest posterior probability (non-overlapping 95% confidence intervals) under the direct approach was then chosen as the best-supported scenario. See further details on the ABC analysis at^90,92^.

### Data availability

The datasets generated during and/or analysed during the current study are available in the DRYAD repository, [10.5061/dryad.t483s94].

## Electronic supplementary material

Supplementary information
