# Supplementary material for: Population genetic signatures of a climate change driven marine range extension
Source: Sci Rep. 2018 Jun 22;8:9558. doi: 10.1038/s41598-018-27351-y (PMC6015011; doi:10.1038/s41598-018-27351-y)
Supplement: Supplementary file 1 — Supplementary information [file 41598_2018_27351_MOESM1_ESM.pdf]

Population genetic signatures of a climate change driven marine range extension

(Supplementary information)

Jorge E. Ramos[1*], Gretta T. Pecl[1,2], Natalie A. Moltschaniwskyj[3], Jayson M. Semmens[1], Carla A. Souza[4], Jan M. Strugnell[4,5]

[1] Institute for Marine and Antarctic Studies, University of Tasmania, Hobart, TAS, 7001, Australia.

[2] Centre for Marine Socioecology, 20 Castray Esplanade, Hobart, TAS, 7001, Australia.

[3] Fisheries Research, Department of Primary Industries, Nelson Bay, NSW, 2315, Australia.

[4] Department of Ecology, Environment and Evolution, La Trobe University, Melbourne, VIC, 3086, Australia.

[5] Present address: Centre for Sustainable Tropical Fisheries and Aquaculture, James Cook University, Townsville, QLD, 4811, Australia.

*Correspondence and requests for materials should be addressed to J.E.R. (email: jeramos@utas.edu.au)

Table S1. Probability for linkage disequilibrium ($P < 0.05$) of corrected data for loci comparison across individuals of *Octopus tetricus* along the east coast of Australia. Comparisons in linkage disequilibrium are indicated in bold text.

Nambucca Heads	Ovul01	Ovul02	Ovul05	Ovul08	Ovul09	Ovul14	Ovul16
Ovul01	-	**0.032**	**0.000**	0.113	0.803	0.752	0.201
Ovul02		-	**0.011**	0.653	0.861	0.086	0.641
Ovul05			-	0.324	**0.014**	0.737	0.123
Ovul08				-	0.715	0.184	0.219
Ovul09					-	0.186	0.659
Ovul14						-	0.777
Ovul16							-

Swansea	Ovul01	Ovul02	Ovul05	Ovul08	Ovul09	Ovul14	Ovul16
Ovul01	-	0.604	**0.000**	0.983	0.908	0.878	0.382
Ovul02		-	0.238	0.244	0.499	0.903	0.875
Ovul05			-	0.844	0.849	0.610	0.405
Ovul08				-	0.408	0.125	0.770
Ovul09					-	0.943	0.594
Ovul14						-	0.268
Ovul16							-

Merimbula	Ovul01	Ovul02	Ovul05	Ovul08	Ovul09	Ovul14	Ovul16
Ovul01	-	0.240	0.094	0.314	0.263	0.941	0.959
Ovul02		-	0.221	0.641	0.060	0.761	0.700
Ovul05			-	0.401	0.153	0.869	0.569
Ovul08				-	0.713	0.699	0.734
Ovul09					-	0.432	0.562
Ovul14						-	0.969
Ovul16							-

Mallacoota	Ovul01	Ovul02	Ovul05	Ovul08	Ovul09	Ovul14	Ovul16
Ovul01	-	**0.037**	0.585	0.881	0.298	0.548	0.778
Ovul02		-	0.157	0.812	0.878	0.612	**0.022**
Ovul05			-	0.448	0.601	0.534	0.982
Ovul08				-	0.489	0.671	0.336
Ovul09					-	**0.008**	0.581
Ovul14						-	0.348
Ovul16							-

Tasmania	Ovul01	Ovul02	Ovul05	Ovul08	Ovul09	Ovul14	Ovul16
Ovul01	-	0.065	**0.000**	**0.000**	**0.017**	0.338	0.569
Ovul02		-	**0.038**	0.212	0.220	0.295	0.937
Ovul05			-	0.278	**0.019**	**0.022**	**0.026**
Ovul08				-	**0.002**	0.082	0.131
Ovul09					-	0.695	0.714
Ovul14						-	0.238
Ovul16							-

Group Ot1	Ovul01	Ovul02	Ovul05	Ovul08	Ovul09	Ovul14	Ovul16
Ovul01	-	**0.009**	**0.001**	0.169	**0.003**	0.430	0.310
Ovul02		-	**0.010**	0.596	0.814	0.954	0.262
Ovul05			-	0.730	**0.032**	0.332	0.235
Ovul08				-	0.093	0.234	0.568
Ovul09					-	0.130	0.421
Ovul14						-	0.769
Ovul16							-
Group Ot2	Ovul01	Ovul02	Ovul05	Ovul08	Ovul09	Ovul14	Ovul16
Ovul01	-	0.052	0.102	**0.000**	0.279	0.143	0.329
Ovul02		-	0.085	0.051	0.314	0.764	0.483
Ovul05			-	0.635	0.203	0.209	0.342
Ovul08				-	0.080	0.395	0.160
Ovul09					-	0.925	0.822
Ovul14						-	0.099
Ovul16							-

Table S2. Probability for linkage disequilibrium (P < 0.05) of uncorrected data for loci comparison across individuals of *Octopus tetricus* along the east coast of Australia. Comparisons in linkage disequilibrium are indicated in bold text.

Nambucca Heads	Ovul01	Ovul02	Ovul05	Ovul08	Ovul09	Ovul14	Ovul16
Ovul01	-	**0.024**	**0.000**	0.072	0.600	0.850	0.097
Ovul02		-	**0.016**	0.659	0.430	0.343	0.424
Ovul05			-	0.348	**0.013**	0.669	0.122
Ovul08				-	0.711	0.705	0.308
Ovul09					-	0.227	0.671
Ovul14						-	0.829
Ovul16							-

Swansea	Ovul01	Ovul02	Ovul05	Ovul08	Ovul09	Ovul14	Ovul16
Ovul01	-	0.778	**0.000**	0.983	0.903	0.861	0.127
Ovul02		-	**0.043**	0.076	0.540	0.678	0.391
Ovul05			-	0.736	0.713	0.279	0.264
Ovul08				-	0.408	0.070	0.799
Ovul09					-	0.966	0.209
Ovul14						-	0.077
Ovul16							-

Merimbula	Ovul01	Ovul02	Ovul05	Ovul08	Ovul09	Ovul14	Ovul16
Ovul01	-	0.112	**0.001**	0.609	0.183	0.929	0.872
Ovul02		-	0.075	0.451	0.179	0.585	0.403
Ovul05			-	0.107	0.068	0.925	0.800
Ovul08				-	0.536	0.637	0.621
Ovul09					-	0.400	0.879
Ovul14						-	0.764
Ovul16							-

Mallacoota	Ovul01	Ovul02	Ovul05	Ovul08	Ovul09	Ovul14	Ovul16
Ovul01	-	**0.001**	0.257	0.981	0.137	0.471	0.180
Ovul02		-	0.462	0.699	0.811	0.558	**0.021**
Ovul05			-	0.390	0.536	0.286	0.854
Ovul08				-	0.627	0.668	0.721
Ovul09					-	**0.008**	0.404
Ovul14						-	0.156
Ovul16							-

Tasmania	Ovul01	Ovul02	Ovul05	Ovul08	Ovul09	Ovul14	Ovul16
Ovul01	-	0.104	**0.000**	**0.000**	**0.008**	0.339	0.275
Ovul02		-	**0.026**	0.125	0.055	0.059	0.681
Ovul05			-	0.148	**0.003**	**0.044**	**0.005**
Ovul08				-	**0.001**	0.134	0.083
Ovul09					-	0.806	0.532
Ovul14						-	**0.030**
Ovul16							-

Group Ot1	Ovul01	Ovul02	Ovul05	Ovul08	Ovul09	Ovul14	Ovul16
Ovul01	-	**0.000**	**0.000**	0.065	**0.004**	0.224	0.177
Ovul02		-	**0.002**	0.301	0.412	0.765	0.070
Ovul05			-	0.732	0.073	0.596	0.164
Ovul08				-	0.075	0.284	0.434
Ovul09					-	0.234	0.054
Ovul14						-	0.571
Ovul16							-

Group Ot2	Ovul01	Ovul02	Ovul05	Ovul08	Ovul09	Ovul14	Ovul16
Ovul01	-	**0.032**	**0.047**	**0.000**	0.395	0.081	0.157
Ovul02		-	0.072	0.052	0.072	0.197	0.593
Ovul05			-	0.292	0.110	**0.029**	0.258
Ovul08				-	0.127	0.392	0.076
Ovul09					-	0.919	0.836
Ovul14						-	**0.015**
Ovul16							-

Table S3. Descriptive statistics for corrected data of *Octopus tetricus* along the east coast of Australia. n – number of individuals genotyped; N_A – number of alleles; N_{PA} – number of private alleles; H_O – observed heterozygosity; H_E – expected heterozygosity; A_R – allelic richness (rarefied to 17 samples); F_{IS} – inbreeding coefficient (F_{IS} values were not significant at $P < 0.05$); P value – significance for Hardy-Weinberg equilibrium ($P < 0.05$). The Group Ot1 is comprised of individuals from all sites, including Ulladulla, Eden and Cape Conran. The distinct Group Ot2 is predominately comprised of individuals from Tasmania (indicated in red in Figs. 1–2 and in Supplementary Fig. S7 online). Descriptive statistics were not presented for Ulladulla, Eden and Cape Conran due to their small sample sizes (n < 17).

Ovul01	Nambucca Heads	Swansea	Merimbula	Mallacoota	Tasmania	Group Ot1	Group Ot2
n	16	29	29	29	57	149	27
N_A	7	8	8	8	11	12	9
N_{PA}	1	1	0	0	0	4	1
H_O	0.750	0.724	0.759	0.690	0.825	0.738	0.852
H_E	0.760	0.731	0.726	0.675	0.858	0.707	0.747
A_R	7.000	6.171	6.984	6.864	8.868	7.325	8.019
F_{IS}	0.014	0.010	-0.046	-0.023	0.039	-0.044	-0.143
P value	0.690	0.054	**0.034**	0.687	**0.000**	**0.010**	0.596

Ovul02	Nambucca Heads	Swansea	Merimbula	Mallacoota	Tasmania	Group Ot1	Group Ot2
n	17	30	29	29	61	152	30
N_A	20	26	27	22	27	38	23
N_{PA}	2	1	1	1	1	16	2
H_O	0.941	0.967	0.897	0.966	0.934	0.921	0.900
H_E	0.954	0.963	0.960	0.955	0.935	0.952	0.949
A_R	19.175	19.328	19.950	17.411	15.677	20.913	19.675
F_{IS}	0.014	-0.004	0.067	-0.012	0.001	0.032	0.052
P value	0.765	0.725	0.129	0.621	0.177	0.112	**0.045**

Ovul05	Nambucca Heads	Swansea	Merimbula	Mallacoota	Tasmania	Group Ot1	Group Ot2
n	16	30	29	29	61	151	30
N_A	3	9	7	8	12	16	6
N_{PA}	0	2	0	2	2	11	1
H_O	0.063	0.400	0.414	0.379	0.656	0.377	0.733
H_E	0.123	0.433	0.414	0.369	0.728	0.360	0.636
A_R	3.000	6.369	5.260	5.778	6.235	5.656	5.601
F_{IS}	0.500	0.078	-0.000	-0.028	0.099	-0.048	-0.157
P value	**0.032**	0.103	0.338	0.624	**0.000**	0.158	0.053

Ovul08	Nambucca Heads	Swansea	Merimbula	Mallacoota	Tasmania	Group Ot1	Group Ot2
n	17	30	29	29	57	151	27
N_A	4	4	5	6	6	7	6
N_{PA}	0	0	1	1	1	2	1
H_O	0.294	0.333	0.379	0.345	0.439	0.344	0.556
H_E	0.271	0.351	0.413	0.313	0.390	0.337	0.489

A_R	3.882	3.801	4.427	4.738	4.294	4.253	5.546
F_{IS}	-0.088	0.051	0.083	-0.102	-0.127	-0.023	-0.139
P value	1.000	0.142	0.462	1.000	1.000	0.620	1.000

Ovul09	Nambucca Heads	Swansea	Merimbula	Mallacoota	Tasmania	Group Ot1	Group Ot2
n	16	30	29	29	49	148	21
N_A	10	14	16	14	19	23	15
N_{PA}	0	1	3	1	3	10	2
H_O	0.875	0.867	0.931	0.862	0.898	0.892	0.905
H_E	0.879	0.892	0.898	0.896	0.899	0.899	0.905
A_R	10.000	11.249	12.510	11.605	12.269	12.906	15.000
F_{IS}	0.005	0.029	-0.037	0.038	0.001	0.007	0.000
P value	0.360	0.853	0.849	0.325	0.845	0.100	0.122

Ovul14	Nambucca Heads	Swansea	Merimbula	Mallacoota	Tasmania	Group Ot1	Group Ot2
n	17	30	29	29	61	152	30
N_A	8	9	6	9	15	14	13
N_{PA}	0	0	0	0	4	4	3
H_O	0.824	0.767	0.793	0.724	0.820	0.770	0.833
H_E	0.843	0.834	0.793	0.754	0.838	0.813	0.859
A_R	7.881	7.637	5.768	6.975	8.782	7.865	10.892
F_{IS}	0.024	0.082	-0.000	0.041	0.022	0.054	0.031
P value	0.912	0.097	0.451	0.098	0.055	**0.000**	0.248

Ovul16	Nambucca Heads	Swansea	Merimbula	Mallacoota	Tasmania	Group Ot1	Group Ot2
n	17	30	29	29	60	152	29
N_A	2	4	4	5	7	7	5
N_{PA}	0	1	0	0	3	4	2
H_O	0.176	0.367	0.379	0.552	0.367	0.401	0.207
H_E	0.166	0.395	0.406	0.549	0.347	0.401	0.256
A_R	2.000	3.523	3.544	4.453	4.168	4.218	4.561
F_{IS}	-0.067	0.073	0.067	-0.004	-0.056	-0.001	0.194
P value	1.000	0.407	0.627	0.173	0.052	0.357	**0.011**

Table S4. Descriptive statistics for uncorrected data of *Octopus tetricus* along the east coast of Australia. n – number of individuals genotyped; N_A – number of alleles; N_{PA} – number of private alleles; H_O – observed heterozygosity; H_E – expected heterozygosity; A_R – allelic richness (rarefied to 17 samples); F_{IS} – inbreeding coefficient (Asterisks indicate significant values at P < 0.05); P value – significance for Hardy-Weinberg equilibrium (P < 0.05). The Group Ot1 is comprised of individuals from all sites, including Ulladulla, Eden and Cape Conran. The distinct Group Ot2 is predominately comprised of individuals from Tasmania. Descriptive statistics were not presented for Ulladulla, Eden and Cape Conran due to their small sample sizes (n < 17).

Ovul01	Nambucca Heads	Swansea	Merimbula	Mallacoota	Tasmania	Group Ot1	Group Ot2
n	16	29	29	29	57	149	27
N_A	6	8	7	7	10	11	8
N_{PA}	1	1	0	0	0	4	1
H_O	0.500	0.724	0.655	0.552	0.719	0.631	0.815
H_E	0.698	0.731	0.702	0.632	0.840	0.678	0.732
A_R	6.000	6.171	6.181	5.949	8.033	6.590	7.241
F_{IS}	0.290	0.010	0.067	0.128	0.145	0.070	-0.115
P value	0.059	0.051	**0.003**	0.229	**0.000**	**0.000**	0.572

Ovul02	Nambucca Heads	Swansea	Merimbula	Mallacoota	Tasmania	Group Ot1	Group Ot2
n	17	30	29	29	61	152	30
N_A	19	25	26	21	26	37	22
N_{PA}	2	1	1	1	1	16	1
H_O	0.706	0.800	0.828	0.759	0.639	0.717	0.700
H_E	0.943	0.961	0.955	0.949	0.933	0.951	0.936
A_R	18.232	19.004	19.197	16.743	15.112	20.699	18.681
F_{IS}	0.257	0.170	0.135	0.204	0.316	0.246	0.255
P value	**0.002**	**0.001**	**0.015**	**0.002**	**0.000**	**0.000**	**0.000**

Ovul05	Nambucca Heads	Swansea	Merimbula	Mallacoota	Tasmania	Group Ot1	Group Ot2
n	16	30	29	29	61	151	30
N_A	3	8	6	7	10	14	5
N_{PA}	0	2	0	2	1	10	1
H_O	0.063	0.267	0.172	0.241	0.295	0.245	0.167
H_E	0.123	0.358	0.284	0.287	0.621	0.265	0.444
A_R	3.000	5.664	4.423	5.065	4.990	4.808	4.612
F_{IS}	0.500	0.259	0.398	0.161	0.527	0.077	0.628
P value	**0.032**	**0.012**	**0.010**	0.192	**0.000**	**0.006**	**0.000**

Ovul08	Nambucca Heads	Swansea	Merimbula	Mallacoota	Tasmania	Group Ot1	Group Ot2
n	17	30	29	29	57	151	27
N_A	4	4	4	6	6	6	6
N_{PA}	0	0	0	1	1	1	1
H_O	0.294	0.333	0.345	0.345	0.439	0.338	0.556
H_E	0.271	0.351	0.387	0.313	0.390	0.331	0.489
A_R	3.882	3.801	3.875	4.738	4.294	4.114	5.546

F_{IS}	-0.088	0.051	0.110	-0.102	-0.127	-0.020	-0.139
P value	1.000	0.143	0.320	1.000	1.000	0.553	1.000

Ovul09	Nambucca Heads	Swansea	Merimbula	Mallacoota	Tasmania	Group Ot1	Group Ot2
n	16	30	29	29	49	148	21
N_A	10	14	16	13	18	22	14
N_{PA}	0	1	3	1	3	10	2
H_O	0.875	0.867	0.931	0.828	0.816	0.858	0.810
H_E	0.879	0.892	0.898	0.895	0.892	0.896	0.894
A_R	10.000	11.249	12.510	11.165	11.546	12.513	14.000
F_{IS}	0.005	0.029	-0.037	0.076	0.086	0.043	0.097
P value	0.348	0.866	0.833	0.219	0.350	0.070	**0.026**

Ovul14	Nambucca Heads	Swansea	Merimbula	Mallacoota	Tasmania	Group Ot1	Group Ot2
n	17	30	29	29	61	152	30
N_A	7	8	6	9	14	13	12
N_{PA}	0	0	0	0	4	4	3
H_O	0.706	0.700	0.793	0.724	0.754	0.743	0.700
H_E	0.824	0.820	0.793	0.754	0.829	0.808	0.842
A_R	6.882	6.851	5.768	6.975	8.269	7.416	10.112
F_{IS}	0.147	0.149	0.000	0.041	0.091	0.081	0.171
P value	0.621	**0.022**	0.447	0.096	**0.006**	**0.000**	**0.006**

Ovul16	Nambucca Heads	Swansea	Merimbula	Mallacoota	Tasmania	Group Ot1	Group Ot2
n	17	30	29	29	60	152	29
N_A	2	3	3	4	6	6	4
N_{PA}	0	1	0	0	3	4	2
H_O	0.176	0.133	0.172	0.379	0.300	0.257	0.138
H_E	0.166	0.239	0.272	0.477	0.307	0.315	0.225
A_R	2.000	2.530	2.551	3.516	3.597	3.396	3.668
F_{IS}	-0.067	0.446	0.369	0.208	0.024	0.186	0.391
P value	1.000	0.057	0.106	**0.016**	**0.009**	**0.001**	**0.002**

Mean	Nambucca Heads	Swansea	Merimbula	Mallacoota	Tasmania	Group Ot1	Group Ot2
n	17	30	29	29	58	151	28
N_A	7	10	10	10	13	16	10
N_{PA}	0	1	1	1	2	7	2
H_O	0.474	0.546	0.557	0.547	0.566	0.541	0.555
H_E	0.558	0.622	0.613	0.615	0.687	0.606	0.652
A_R	7.142	7.896	7.786	7.736	7.977	8.505	9.123
F_{IS}	0.181*	0.143*	0.128*	0.123*	0.184*	0.121*	0.144*
P value	**0.000**	**0.000**	**0.012**	**0.000**	**0.000**	**0.000**	**0.000**

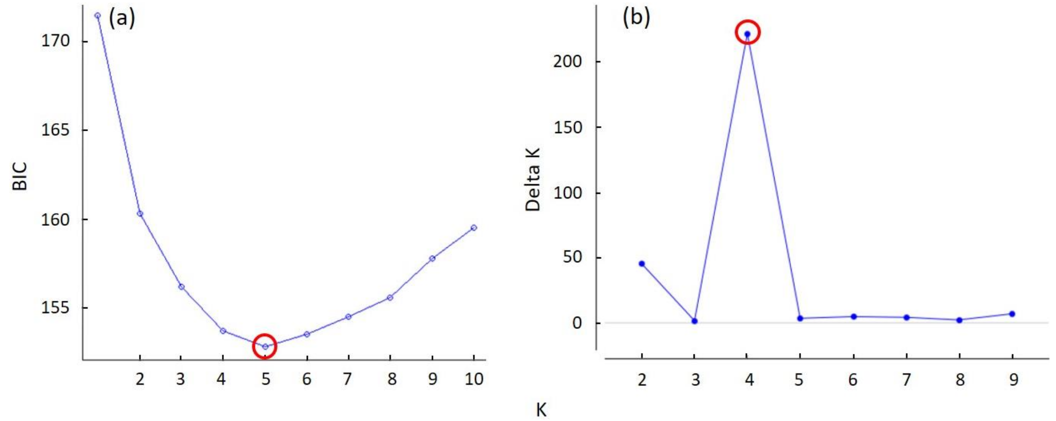

Figure S5. Detection of the number of clusters (K) that best fit corrected data using the (a) Discriminant Analysis of Principal Components (DAPC) and (b) Structure analyses for *Octopus tetricus* from the east coast of Australia. The chosen number of clusters is indicated by the greatest changes in Bayesian Information Criterion (BIC)[1] and Delta K[2] (red circles).

Table S6. Percentage (%) contribution based on uncorrected data of *Octopus tetricus* individuals from the east coast of Australia to assigned clusters estimated in DAPC and Structure. DAPC – Discriminant Analysis of Principal Components. Nambucca Heads (n = 17); Swansea (n = 30); Ulladulla (n = 5); Merimbula (n = 29); Eden (n = 8); Mallacoota (n = 29); Cape Conran (n = 3); Tasmania (n = 61).

Site	DAPC					Structure				
	1	2	3	4	5	1	2	3	4	5
Nambucca Heads	24	29	41	6	0	18	24	35	24	0
Swansea	33	20	30	13	3	27	20	33	13	7
Ulladulla	0	60	40	0	0	0	0	60	40	0
Merimbula	21	31	34	14	0	14	21	34	24	7
Eden	0	63	13	25	0	25	38	0	38	0
Mallacoota	21	3	34	41	0	28	14	21	34	3
Cape Conran	0	0	100	0	0	33	33	0	33	0
Tasmania	18	8	21	15	38	16	23	3	20	38

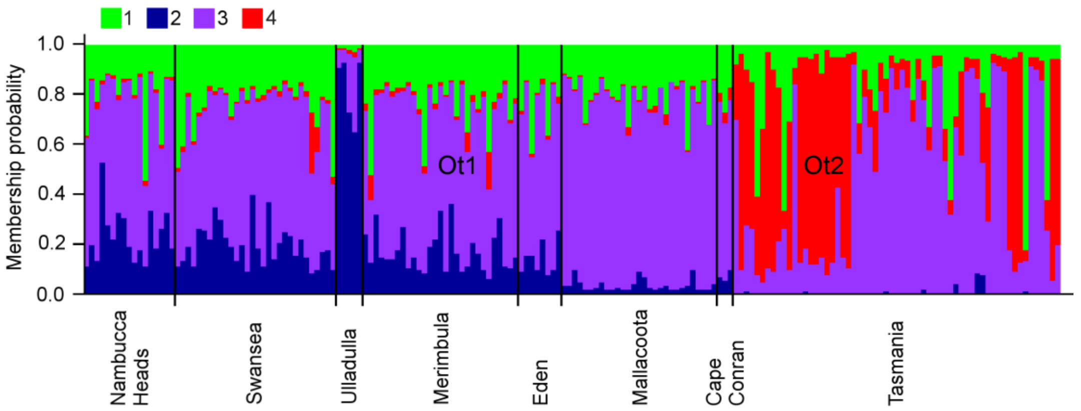

Figure S7. Individual assignment probabilities of *Octopus tetricus* from the east coast of Australia (columns) to identified clusters (K = 4) inferred with Structure and using corrected data. Sites of collection are separated by thick solid lines. The historical distribution zone is comprised of Nambucca Heads, Swansea, Ulladulla, Merimbula, and Eden. The range extension zone is comprised of Mallacoota, Cape Conran, and north-eastern Tasmania. Ot1 – common group comprised of individuals from along the east coast of Australia and Tasmania; Ot2 – distinct group predominately comprised of individuals from Tasmania (indicated in red).

Phylogenetic analysis

Partial sequences of 650 bp of the mitochondrial gene Cytochrome Oxidase subunit I (COI) were targeted in 25 µL reactions comprised of 12.5 µL MyTaq Red Mix (Bioline), 0.5 µl forward primer LCO1490 (10 µM), 0.5 µL reverse primer HCO2198 (10 µM)[3], 9.5 µL ddH_2O, and 2 µL DNA (10–30 ng/µL). Reaction conditions included a denaturation step of 95°C for 2 min, followed by 35 cycles of 95°C for 30 s, 48°C for 30 s, and 72°C for 30 s, and a final extension step of 72°C for 5 min. PCR products were sequenced by Macrogen Inc. (Seoul, Korea). A phylogenetic tree was constructed with a set of subsamples to corroborate that individuals of the different groups detected from population structure analyses were in fact *O. tetricus* and not representative of a cryptic species. The phylogenetic analysis also included GenBank sequences of closely related species, i.e. *O.* cf. *tetricus* from Western Australia and *O. vulgaris* from different areas[4,5], with *O. mimus* as the outgroup species (see GenBank sequence accession numbers in Supplementary Fig. S8 online). jModelTest v. 0.1.1[6] was used to carry out statistical selection of best-fit models on the basis of goodness of fit measures using the Akaike Information Criterion[7]. Topologies were constructed via Maximum Likelihood using PhyML v. 3.1[8]. Searches were undertaken and model parameter values were estimated; 1,000 bootstrap replicates were used to measure the strength of support for internal nodes. Bayesian marginal posterior probabilities were calculated using MrBayes v. 3.2[9]. Parameters of the model were estimated; random starting trees were used and the analysis was run with 15 million generations, sampling the Markov chain every 1,000 generations. Convergence of the Markov chain and appropriate burnin length were assessed using Tracer v. 1.6[10].

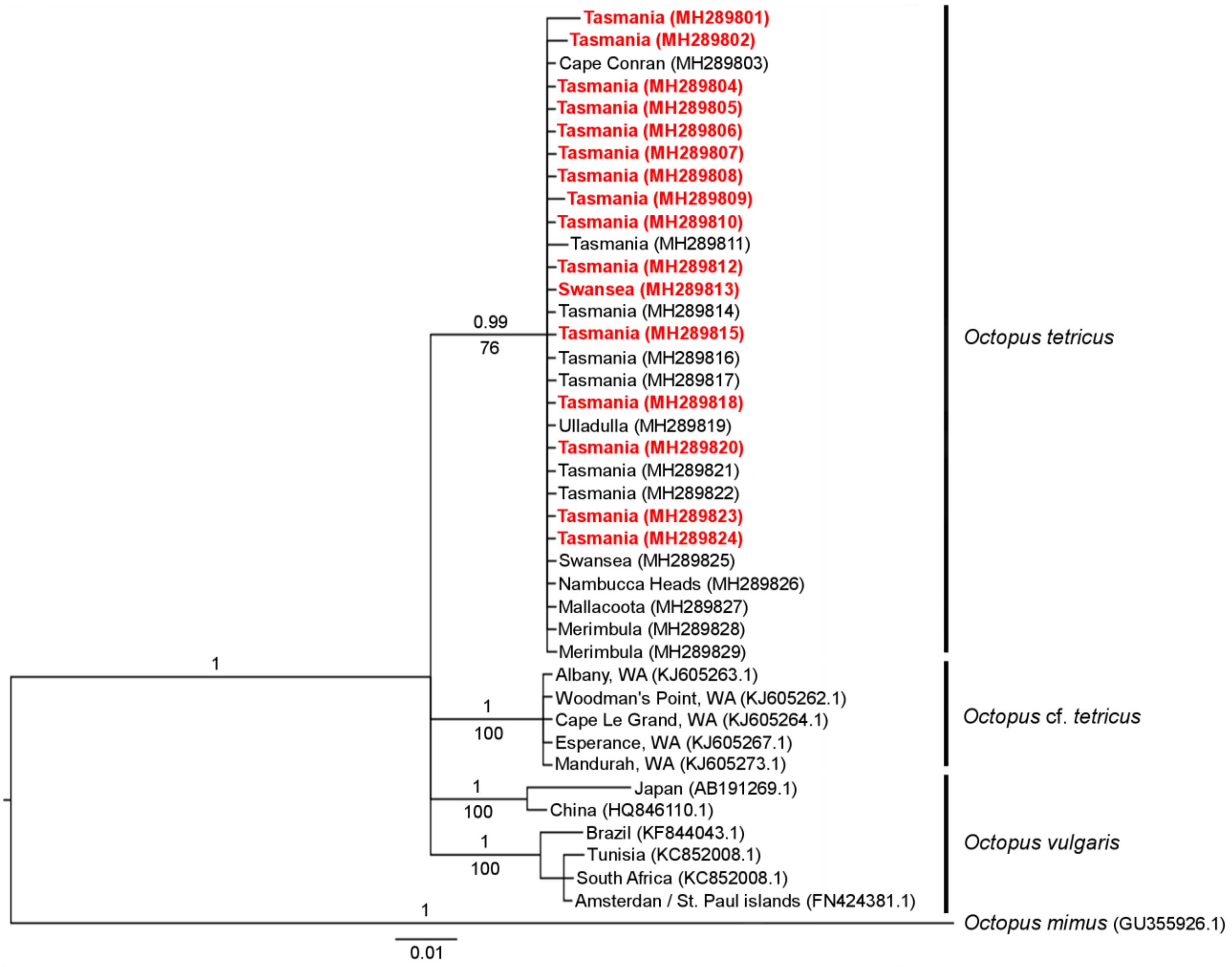

Figure S8. Bayesian and Maximum Likelihood topologies of *Octopus tetricus* along eastern Australia compared to genetically close species *Octopus* cf. *tetricus* and *Octopus vulgaris*. Phylogenetic analysis is based on the mitochondrial gene COI. Bayesian posterior probabilities and maximum likelihood bootstrap values are indicated above and below the branches respectively. Outgroup is *Octopus mimus*. Node labels indicate the location of each individual. *Octopus tetricus* individuals belonging to Group Ot1 (n = 13) are in normal text. *Octopus tetricus* individuals belonging to Group Ot2 (n = 16) are in bold red text. WA – Western Australia. GenBank accession numbers are indicated in parentheses.

Table S9. F_{ST} among collection sites for uncorrected data of *Octopus tetricus* along the east coast of Australia. F_{ST} values are indicated below the diagonal. P values are indicated above the diagonal. Bold indicates significant values at $P < 0.05$. F_{ST} was not estimated for Ulladulla, Eden and Cape Conran due to their small sample sizes ($n < 17$).

Site	Nambucca Heads	Swansea	Merimbula	Mallacoota	Tasmania
Nambucca Heads	-	0.7158	0.9725	**0.0398**	**0.0460**
Swansea	-0.0016	-	0.6756	**0.0184**	**0.0127**
Merimbula	-0.0099	-0.0012	-	**0.0061**	**0.0096**
Mallacoota	**0.0223**	**0.0193**	**0.0232**	-	**<0.001**
Tasmania	**0.0157**	**0.0136**	**0.0150**	**0.0302**	**-**

Table S10. Association between allelic frequencies and year, and depth of collection of *Octopus tetricus* along the east coast of Australia. The Spearman's correlation was used to examine the association between variables. $P_{crit} < 0.05$.

Correlation	Uncorrected data		Corrected data	
	r_s	P-value	r_s	P-value
Ovul01 - year	0.239	**0.001**	0.231	**0.002**
Ovul02 - year	-0.058	0.441	-0.071	0.343
Ovul05 - year	0.247	**0.001**	0.112	0.134
Ovul08 - year	0.147	**0.047**	0.148	**0.047**
Ovul09 - year	0.026	0.728	0.016	0.827
Ovul14 - year	0.004	0.954	0.009	0.909
Ovul16 - year	-0.029	0.699	0.049	0.513
Ovul01 - depth	-0.234	**0.002**	-0.230	**0.002**
Ovul02 - depth	0.069	0.357	0.086	0.251
Ovul05 - depth	-0.324	**< 0.001**	-0.176	**0.018**
Ovul08 - depth	-0.138	0.063	-0.145	0.051
Ovul09 - depth	-0.028	0.713	-0.018	0.805
Ovul14 - depth	-0.019	0.803	-0.011	0.879
Ovul16 - depth	-0.015	0.837	-0.096	0.200

Table S11. Inferred (posterior mean) migration rates from uncorrected data for *Octopus tetricus* between collection sites along the east coast of Australia. Italicised values indicate self-recruitment. Values in parentheses are the standard deviations of the marginal posterior distribution for each estimate. Left column indicates where migrants travelled to; top row indicates where migrants originated from.

Site	Nambucca Heads	Swansea	Ulladulla	Merimbula	Eden	Mallacoota	Cape Conran	Tasmania
Nambucca Heads	*0.681 (0.013)*	0.014 (0.013)	0.014 (0.013)	0.222 (0.032)	0.014 (0.013)	0.014 (0.013)	0.014 (0.013)	0.030 (0.021)
Swansea	0.009 (0.009)	*0.676 (0.009)*	0.009 (0.009)	0.246 (0.026)	0.009 (0.009)	0.009 (0.009)	0.009 (0.009)	0.034 (0.018)
Ulladulla	0.026 (0.024)	0.026 (0.024)	*0.694 (0.025)*	0.153 (0.044)	0.025 (0.023)	0.026 (0.024)	0.026 (0.024)	0.026 (0.024)
Merimbula	0.009 (0.009)	0.009 (0.009)	0.009 (0.009)	*0.915 (0.024)*	0.009 (0.009)	0.009 (0.009)	0.009 (0.009)	0.032 (0.017)
Eden	0.021 (0.020)	0.021 (0.020)	0.021 (0.020)	0.186 (0.040)	*0.687 (0.020)*	0.021 (0.020)	0.021 (0.020)	0.021 (0.020)
Mallacoota	0.009 (0.009)	0.009 (0.009)	0.009 (0.009)	0.266 (0.023)	0.009 (0.009)	*0.676 (0.009)*	0.009 (0.009)	0.013 (0.012)
Cape Conran	0.030 (0.028)	0.030 (0.028)	0.030 (0.028)	0.120 (0.046)	0.030 (0.028)	0.030 (0.028)	*0.699 (0.029)*	0.030 (0.028)
Tasmania	0.005 (0.005)	0.005 (0.005)	0.005 (0.005)	0.179 (0.022)	0.005 (0.005)	0.005 (0.005)	0.005 (0.005)	*0.792 (0.021)*

Table S12. Heterozygosity excess tests results performed using uncorrected data to detect genetic bottleneck on *Octopus tetricus* along the east coast of Australia. The Group Ot1 is comprised of individuals from all sites. The distinct Group Ot2 is predominately comprised of individuals from Tasmania. Values for sites with ≥17 samples are presented only. SMM – Stepwise Mutation Model; TPM – Two-Phase Mutation Model. IAM – Infinite Allele Model; i – "Sign test"; ii – "2-tailed Wilcoxon sign rank test"; iii – "Mode-shift test". L-shaped – no bottleneck effect detected. Significance at $P < 0.05$, with significant bottleneck effect indicated in bold.

	SMM		TPM		IAM		
Site/Group	i	ii	i	ii	i	ii	iii
Nambucca Heads	0.113	**0.039**	0.120	0.297	0.362	0.578	L-shaped
Swansea	0.101	**0.039**	0.283	0.297	0.612	0.813	L-shaped
Merimbula	**0.019**	**0.023**	**0.020**	0.109	0.617	0.938	L-shaped
Mallacoota	**0.021**	**0.023**	0.090	0.078	0.589	0.813	L-shaped
Tasmania	**0.002**	**0.008**	**0.027**	0.055	0.582	0.813	L-shaped
Group Ot1	**0.002**	**0.008**	**0.022**	**0.040**	0.291	0.688	L-shaped
Group Ot2	**0.002**	**0.008**	**0.002**	**0.008**	0.593	0.578	L-shaped

Table S13. Model specification, prior distributions for demographic parameters and locus-specific mutation model parameters used in the Approximate Bayesian Computation analysis to select the most likely population topology for *Octopus tetricus* along the east coast of Australia. N1/N2/N3/N4 – current effective population size of each geographic cluster; t – time in generations. N5 and N6 refers to the past change in effective population size that led to the current population size of N2 and N3, respectively; SNI – Single Nucleotide Insertion.

**Demographic parameter**	**Distribution**	**Minimum**	**Maximum**
N1	Uniform	10	10,000
N2	Uniform	10	10,000
N3	Uniform	10	10,000
N4	Uniform	10	10,000
N5	Uniform	10	10,000
N6	Uniform	10	10,000
t_i (conditions: $t_1<t_2<t_3<t_4<t_5$)	Uniform	10	10,000
Admixture rate	Uniform	0.1	0.9
**Microsatellite mutation parameter**	**Distribution**	**Minimum**	**Maximum**
Mean mutation rate	Gamma	0.0001	0.001
Mean coefficient P	Gamma	0	0
Mean SNI rate	Log-uniform	1.0^{-8}	1.0^{-5}

Table S14. Model checking procedure to select the most likely population topology for *Octopus tetricus* along the east coast of Australia using the Approximate Bayesian Computation analysis. The probability *Prob*.($S_{simul.}$ < $S_{obs.}$) given for each summary statistic was calculated from 1,000 virtual datasets simulated from the posterior distributions of parameters obtained under the focused scenario. The star indicates proportions of simulated data (considering the total reference table) with significant deviations below or above the value of the observed dataset. An increasing number of marginal summary statistics (noted by *, **, *** = p-values < 0.05, < 0.01 and < 0.001, respectively) suggest that scenario 4 is better supported than other scenarios. One-sample, two-sample and admixture summary statistics were used to evaluate how well the scenario and priors of parameters fit the data in the summary statistics. NAL – mean number of alleles across loci; HET – mean genic diversity across loci; VAR – mean allele size variance across loci; MGW – mean Garcia-Williamson index; N2P – mean number of alleles across loci (two samples); H2P – mean genic diversity across loci (two sample); V2P – mean allele size variance across loci (two samples); FST – F_{ST} statistics; LIK – mean index of classification (two samples); DAS – shared allele distance (two samples); DM2 – $(\delta\mu)^2$ distance between two samples; AML – admixture maximum likelihood[11]. The numbers after each statistic refer to the sub-groups: Sub-group 1 – Nambucca Heads, Swansea, and Merimbula; Sub-group 2 – Mallacoota; Sub-group 3 – Ot1 Tasmania; Sub-group 4 – distinct Group Ot2 predominately comprised of individuals from Tasmania (indicated in red in Figs. 1–2).

		Prob. ($S_{simul.}$ < $S_{obs.}$)				
Statistics	Observed	Scenario 1	Scenario 2	Scenario 3	Scenario 4	Scenario 5
NAL_1_1	15.0000	0.7480	0.6905	0.8335	0.7900	0.7665
NAL_1_2	10.5714	0.3725	0.2825	0.5085	0.4960	0.2600
NAL_1_3	11.0000	0.3785	0.3060	0.3710	0.4625	0.4310
NAL_1_4	10.2857	0.6790	0.6940	0.6480	0.7045	0.6110
HET_1_1	0.6384	0.0000 (***)	0.0000 (***)	0.0000 (***)	0.0020 (**)	0.0000 (***)
HET_1_2	0.6385	0.0010 (***)	0.0010 (***)	0.0005 (***)	0.0010 (***)	0.0000 (***)
HET_1_3	0.6717	0.0030 (**)	0.0010 (***)	0.0020 (**)	0.0030 (**)	0.0035 (**)
HET_1_4	0.7051	0.0425 (*)	0.0660	0.0340 (*)	0.0545	0.0310 (*)
VAR_1_1	5.1877	0.1320	0.0790	0.2025	0.1405	0.1290
VAR_1_2	6.5469	0.2340	0.1840	0.3450	0.2960	0.2055
VAR_1_3	7.6420	0.3590	0.2740	0.3640	0.4055	0.3680
VAR_1_4	8.0216	0.5210	0.4365	0.5650	0.5450	0.4735
MGW_1_1	1.8261	1.0000 (***)	1.0000 (***)	1.0000 (***)	1.0000 (***)	1.0000 (***)
MGW_1_2	1.1935	0.9635 (*)	0.9770 (*)	0.9615 (*)	0.9825 (*)	0.9580 (*)
MGW_1_3	1.1324	0.8545	0.9235	0.8615	0.9005	0.8910
MGW_1_4	1.0746	0.8920	0.9270	0.8470	0.8960	0.8775
N2P_1_1&2	16.7143	0.8525	0.7330	0.8495	0.8125	0.6280
N2P_1_1&3	17.0000	0.8290	0.4570	0.7890	0.8575	0.6095
N2P_1_1&4	16.7143	0.5455	0.5620	0.6360	0.6395	0.5765
N2P_1_2&3	13.5714	0.5135	0.1630	0.5480	0.6380	0.4410
N2P_1_2&4	14.1429	0.3955	0.3840	0.4515	0.4720	0.3840
N2P_1_3&4	14.4286	0.4045	0.5010	0.5040	0.4580	0.5000
H2P_1_1&2	0.6419	0.0000 (***)	0.0010 (***)	0.0000 (***)	0.0010 (***)	0.0000 (***)
H2P_1_1&3	0.6503	0.0000 (***)	0.0000 (***)	0.0000 (***)	0.0020 (**)	0.0000 (***)
H2P_1_1&4	0.6898	0.0000 (***)	0.0000 (***)	0.0010 (***)	0.0020 (**)	0.0020 (**)
H2P_1_2&3	0.6577	0.0010 (***)	0.0000 (***)	0.0000 (***)	0.0020 (**)	0.0000 (***)

H2P_1_2&4	0.7269	0.0010 (***)	0.0010 (***)	0.0030 (**)	0.0070 (**)	0.0030 (**)
H2P_1_3&4	0.7290	0.0025 (**)	0.0030 (**)	0.0065 (**)	0.0055 (**)	0.0055 (**)
V2P_1_1&2	5.5302	0.1560	0.0990	0.2240	0.1640	0.1100
V2P_1_1&3	5.8945	0.1810	0.0740	0.2395	0.2015	0.1230
V2P_1_1&4	6.4166	0.1630	0.1020	0.2130	0.1830	0.1540
V2P_1_2&3	7.0868	0.2750	0.1270	0.3360	0.3265	0.2700
V2P_1_2&4	7.9790	0.2660	0.2030	0.3215	0.3110	0.2710
V2P_1_3&4	8.3774	0.2910	0.2940	0.3760	0.3360	0.3510
FST_1_1&2	0.0136	0.7695	0.3760	0.1880	0.1345	0.0090 (**)
FST_1_1&3	0.0094	0.2715	0.0000 (***)	0.0335 (*)	0.1955	0.0000 (***)
FST_1_1&4	0.1364	0.9060	0.9070	0.9025	0.9085	0.9270
FST_1_2&3	0.0058	0.0765	0.0000 (***)	0.1200	0.1875	0.0740
FST_1_2&4	0.1486	0.9255	0.9290	0.9225	0.9135	0.9790 (*)
FST_1_3&4	0.1126	0.7130	0.8650	0.8815	0.7370	0.8885
LIK_1_1&2	1.5444	0.0660	0.0210 (*)	0.0340 (*)	0.0250 (*)	0.0010 (***)
LIK_1_1&3	1.5562	0.0510	0.0000 (***)	0.0350 (*)	0.0515	0.0000 (***)
LIK_1_1&4	2.1124	0.0060 (**)	0.0060 (**)	0.0120 (*)	0.0100 (**)	0.0095 (**)
LIK_1_2&1	1.4530	0.0615	0.0145 (*)	0.0290 (*)	0.0200 (*)	0.0000 (***)
LIK_1_2&3	1.4292	0.0110 (*)	0.0000 (***)	0.0290 (*)	0.0410 (*)	0.0060 (**)
LIK_1_2&4	2.1484	0.0050 (**)	0.0080 (**)	0.0120 (*)	0.0150 (*)	0.0230 (*)
LIK_1_3&1	1.5172	0.0620	0.0000 (***)	0.0130 (*)	0.0640	0.0000 (***)
LIK_1_3&2	1.4752	0.0200 (*)	0.0000 (***)	0.0180 (*)	0.0430 (*)	0.0295 (*)
LIK_1_3&4	2.0914	0.0060 (**)	0.0190 (*)	0.0195 (*)	0.0090 (**)	0.0195 (*)
LIK_1_4&1	2.2451	0.0405 (*)	0.0720	0.0305 (*)	0.0570	0.0420 (*)
LIK_1_4&2	2.5949	0.1540	0.2315	0.1330	0.1780	0.3705
LIK_1_4&3	2.4255	0.0885	0.3235	0.3215	0.1070	0.2370
DAS_1_1&2	0.3526	1.0000 (***)	0.9990 (***)	1.0000 (***)	0.9990 (***)	1.0000 (***)
DAS_1_1&3	0.3388	1.0000 (***)	1.0000 (***)	1.0000 (***)	0.9980 (**)	1.0000 (***)
DAS_1_1&4	0.2250	0.9990 (***)	0.9980 (**)	0.9970 (**)	0.9930 (**)	0.9960 (**)
DAS_1_2&3	0.3412	0.9990 (***)	1.0000 (***)	1.0000 (***)	0.9980 (**)	1.0000 (***)
DAS_1_2&4	0.2109	0.9980 (**)	0.9970 (**)	0.9930 (**)	0.9885 (*)	0.9900 (**)
DAS_1_3&4	0.2243	0.9970 (**)	0.9950 (**)	0.9900 (**)	0.9920 (**)	0.9910 (**)
DM2_1_1&2	0.0448	0.0290 (*)	0.0145 (*)	0.0085 (**)	0.0025 (**)	0.0000 (***)
DM2_1_1&3	0.2072	0.2330	0.0010 (***)	0.0970	0.2070	0.0030 (**)
DM2_1_1&4	3.5305	0.2620	0.3310	0.2855	0.2950	0.3085
DM2_1_2&3	0.0834	0.0325 (*)	0.0000 (***)	0.0255 (*)	0.0395 (*)	0.0205 (*)
DM2_1_2&4	3.3469	0.2365	0.3125	0.2510	0.2660	0.4050
DM2_1_3&4	2.5898	0.1635	0.3580	0.2675	0.1920	0.3185
AML_1_3&1&2	0.5010	0.0465 (*)	0.1785	0.6020	0.4265	0.9910 (**)

Table S15. Original parameter estimation and statistics (mean, median, mode and quantiles) of the posterior distribution for five scenarios examined to select the most likely population topology for *Octopus tetricus* along the east coast of Australia using the Approximate Bayesian Computation analysis. N1/N2/N3/N4/N5/N6 – effective population size of each geographic cluster; t – time in generations. Âµmic – mean mutation rate; snimic – mean Single Nucleotide Insertion/Deletion rate.

Parameter	mean	median	mode	q025	q050	q250	q750	q950	q975
**Scenario 1**									
N1	9.31E+03	9.44E+03	9.63E+03	7.93E+03	8.26E+03	9.07E+03	9.70E+03	9.93E+03	9.97E+03
N2	5.76E+03	5.78E+03	6.47E+03	1.72E+03	2.23E+03	4.05E+03	7.47E+03	9.34E+03	9.69E+03
N3	7.81E+03	8.03E+03	8.65E+03	4.47E+03	5.05E+03	6.90E+03	8.97E+03	9.80E+03	9.90E+03
N4	6.44E+03	6.47E+03	6.49E+03	2.74E+03	3.21E+03	5.11E+03	7.91E+03	9.47E+03	9.72E+03
t1	1.77E+02	1.47E+02	1.29E+02	2.77E+01	3.96E+01	9.21E+01	2.22E+02	4.09E+02	5.01E+02
t2	3.45E+02	2.67E+02	2.00E+02	8.12E+01	9.90E+01	1.78E+02	4.14E+02	8.19E+02	1.07E+03
t3	6.24E+03	6.25E+03	6.55E+03	2.58E+03	3.08E+03	4.81E+03	7.70E+03	9.45E+03	9.73E+03
Âµmic_1	6.61E-04	6.63E-04	6.68E-04	3.44E-04	3.89E-04	5.41E-04	7.82E-04	9.41E-04	9.72E-04
snimic_1	9.90E-06	1.00E-05	1.00E-05	8.76E-06	9.56E-06	1.00E-05	1.00E-05	1.00E-05	1.00E-05
**Scenario 2**									
N1	8.90E+03	9.07E+03	9.38E+03	6.91E+03	7.32E+03	8.49E+03	9.47E+03	9.88E+03	9.94E+03
N2	7.35E+03	7.61E+03	8.02E+03	3.27E+03	4.07E+03	6.24E+03	8.71E+03	9.72E+03	9.87E+03
N3	8.79E+03	8.98E+03	9.38E+03	6.48E+03	7.01E+03	8.31E+03	9.49E+03	9.89E+03	9.94E+03
N4	5.12E+03	4.90E+03	3.82E+03	1.63E+03	1.95E+03	3.45E+03	6.68E+03	9.01E+03	9.48E+03
t1	4.44E+02	3.70E+02	2.09E+02	5.77E+01	8.65E+01	2.18E+02	5.68E+02	1.07E+03	1.33E+03
t2	3.01E+03	2.71E+03	2.18E+03	8.32E+02	1.06E+03	1.86E+03	3.83E+03	6.04E+03	6.80E+03
t3	4.47E+03	4.10E+03	3.21E+03	1.26E+03	1.52E+03	2.83E+03	5.80E+03	8.63E+03	9.27E+03
Âµmic_1	7.48E-04	7.56E-04	7.63E-04	4.42E-04	4.96E-04	6.48E-04	8.62E-04	9.75E-04	9.93E-04
snimic_1	9.76E-06	1.00E-05	1.00E-05	6.84E-06	8.26E-06	1.00E-05	1.00E-05	1.00E-05	1.00E-05
**Scenario 3**									
N1	8.80E+03	8.97E+03	9.19E+03	6.66E+03	7.10E+03	8.34E+03	9.46E+03	9.89E+03	9.94E+03
N2	6.80E+03	6.90E+03	7.10E+03	3.04E+03	3.66E+03	5.53E+03	8.20E+03	9.56E+03	9.77E+03
N3	5.70E+03	5.67E+03	5.43E+03	1.53E+03	2.00E+03	4.04E+03	7.41E+03	9.39E+03	9.68E+03
N4	7.04E+03	7.14E+03	6.75E+03	3.50E+03	4.04E+03	5.87E+03	8.35E+03	9.61E+03	9.82E+03
t1	2.49E+02	1.97E+02	1.05E+02	2.61E+01	3.88E+01	1.11E+02	3.25E+02	6.37E+02	7.93E+02
r1	8.49E-01	8.69E-01	8.90E-01	6.42E-01	7.35E-01	8.44E-01	8.85E-01	8.97E-01	8.98E-01
t2	5.84E+02	4.49E+02	2.63E+02	1.28E+02	1.56E+02	2.92E+02	6.97E+02	1.43E+03	1.88E+03
t3	6.72E+03	6.81E+03	6.67E+03	2.98E+03	3.48E+03	5.39E+03	8.21E+03	9.56E+03	9.78E+03
Âµmic_1	6.24E-04	6.19E-04	6.16E-04	3.12E-04	3.58E-04	4.99E-04	7.45E-04	9.13E-04	9.54E-04
snimic_1	9.92E-06	1.00E-05	1.00E-05	8.95E-06	9.66E-06	1.00E-05	1.00E-05	1.00E-05	1.00E-05

**Scenario 4**									
N1	8.82E+03	8.98E+03	9.33E+03	6.75E+03	7.23E+03	8.39E+03	9.43E+03	9.87E+03	9.93E+03
N2	7.22E+03	7.36E+03	7.73E+03	3.74E+03	4.28E+03	6.12E+03	8.49E+03	9.63E+03	9.81E+03
N3	5.52E+03	5.44E+03	4.42E+03	1.43E+03	1.91E+03	3.80E+03	7.26E+03	9.36E+03	9.68E+03
N4	6.76E+03	6.83E+03	6.79E+03	3.19E+03	3.71E+03	5.53E+03	8.12E+03	9.54E+03	9.75E+03
t1	9.95E+01	7.77E+01	5.26E+01	1.63E+01	2.10E+01	4.71E+01	1.25E+02	2.48E+02	3.10E+02
r1	6.11E-01	6.41E-01	6.80E-01	1.73E-01	2.34E-01	5.00E-01	7.54E-01	8.66E-01	8.82E-01
t2	5.37E+02	3.92E+02	2.40E+02	1.07E+02	1.33E+02	2.52E+02	6.29E+02	1.39E+03	1.91E+03
t3	4.89E+03	4.58E+03	3.26E+03	1.38E+03	1.69E+03	3.09E+03	6.54E+03	9.07E+03	9.52E+03
Âµmic_1	6.94E-04	6.98E-04	6.85E-04	3.80E-04	4.27E-04	5.83E-04	8.11E-04	9.51E-04	9.82E-04
snimic_1	9.93E-06	1.00E-05	1.00E-05	9.13E-06	9.78E-06	1.00E-05	1.00E-05	1.00E-05	1.00E-05
**Scenario 5**									
N1	9.27E+03	9.39E+03	9.74E+03	7.84E+03	8.20E+03	9.01E+03	9.68E+03	9.92E+03	9.96E+03
N2	5.24E+03	5.04E+03	3.95E+03	1.14E+03	1.66E+03	3.46E+03	7.06E+03	9.22E+03	9.59E+03
N3	8.03E+03	8.25E+03	8.86E+03	4.92E+03	5.52E+03	7.21E+03	9.07E+03	9.81E+03	9.90E+03
N4	7.09E+03	7.22E+03	7.80E+03	3.67E+03	4.14E+03	5.94E+03	8.38E+03	9.62E+03	9.77E+03
N5	4.97E+03	4.90E+03	4.85E+03	3.97E+02	6.70E+02	2.57E+03	7.36E+03	9.47E+03	9.73E+03
N6	5.03E+03	4.98E+03	6.67E+03	3.15E+02	5.86E+02	2.55E+03	7.45E+03	9.48E+03	9.70E+03
t1	1.66E+02	1.23E+02	5.50E+01	1.86E+01	2.58E+01	6.70E+01	2.13E+02	4.50E+02	5.72E+02
r1	3.03E-01	2.66E-01	1.60E-01	1.11E-01	1.23E-01	1.87E-01	3.79E-01	6.23E-01	7.08E-01
t2	8.04E+02	6.52E+02	3.43E+02	1.46E+02	1.89E+02	4.03E+02	1.01E+03	1.96E+03	2.42E+03
t3	2.04E+03	1.76E+03	1.46E+03	4.88E+02	6.08E+02	1.14E+03	2.59E+03	4.58E+03	5.34E+03
t4	2.58E+03	2.19E+03	1.52E+03	5.91E+02	7.37E+02	1.40E+03	3.35E+03	5.84E+03	6.66E+03
t5	5.82E+03	5.70E+03	4.64E+03	1.95E+03	2.29E+03	4.04E+03	7.65E+03	9.45E+03	9.72E+03
Âµmic_1	6.63E-04	6.66E-04	6.74E-04	3.45E-04	3.93E-04	5.47E-04	7.81E-04	9.25E-04	9.66E-04
snimic_1	9.69E-06	1.00E-05	1.00E-05	6.39E-06	7.86E-06	1.00E-05	1.00E-05	1.00E-05	1.00E-05

Table S16. Posterior probabilities for the population topology model choice for *Octopus tetricus* along the east coast of Australia using the direct approach of the Approximate Bayesian Computation analysis. * – Type-I error rate; † – Type-II error rate.

	**Scenario 1**	**Scenario 2**	**Scenario 3**	**Scenario 4**	**Scenario 5**
**Posterior Probability**	0.294	0.100	0.206	0.332	0.068
**95% CI**	[0.0000–0.6933]	[0.0000–0.3630]	[0.0000–0.5605]	[0.0000–0.7448]	[0.0000–0.2887]
**Scenario 1**	44.4%	17.7%	7.8%	12.8%*	6.90%
**Scenario 2**	21.3%	59.6%	13.1%	3.6%*	9.9%
**Scenario 3**	6.8%	11.0%	46.0%	12%*	17.3%
**Scenario 4**	16.5%†	4.1%†	15.8%†	58.7%	12.1% †
**Scenario 5**	11.0%	7.6%	17.3%	12.9%*	53.8%

Table S17. Model specification, prior distributions for demographic parameters and locus-specified mutation model parameters used in the Approximate Bayesian Computation analysis to refine changes in population size (genetic bottlenecks) and divergence times for *Octopus tetricus* along the east coast of Australia. N1/N2/N3/N4 – effective population size of each geographic cluster; t – time in generations; SNI – Single Nucleotide Insertion. Conditions: t3>t2>t1b>t1>t0b; N4≥N4b; N1b≥N1; N2b≤N2.

**Demographic parameter**	**Distribution**	**Minimum**	**Maximum**
N1	Uniform	10	10,000
N2	Uniform	10	10,000
N3	Uniform	10	10,000
N4	Uniform	10	10,000
N1b	Uniform	10	10,000
N2b	Uniform	10	10,000
N4b	Uniform	10	400
t0b	Uniform	10	60
t1	Uniform	10	100
t1b	Uniform	10	10,000
t2	Uniform	10	200
t3	Uniform	10	2,000
Admixture rate	Uniform	0.1	0.9
**Microsatellite mutation parameter**	**Distribution**	**Minimum**	**Maximum**
Mean mutation rate	Gamma	0.0001	0.001
Mean coefficient P	Gamma	0	0
Mean SNI rate	Log-uniform	1.0^{-8}	1.0^{-5}

Table S18. Model checking procedure to refine changes in population size (genetic bottlenecks) and divergence times for *Octopus tetricus* along the east coast of Australia examined using the Approximate Bayesian Computation analysis. The probability *Prob.*($S_{simul.} < S_{obs.}$) given for each summary statistic was calculated from 1,000 virtual datasets simulated from the posterior distributions of parameters obtained under the focused scenario. The star indicates proportions of simulated data (considering the total reference table) with significant deviations below or above the value of the observed dataset. An increasing number of marginal summary statistics (noted by *, **, *** = p-values < 0.05, < 0.01 and < 0.001, respectively) suggest that scenario 6 is better supported than the other two scenarios. One-sample, two-sample and admixture summary statistics were used to evaluate how well the scenario and priors of parameters fit the data in the summary statistics. NAL – mean number of alleles across loci; HET – mean genic diversity across loci; VAR – mean allele size variance across loci; MGW – mean Garcia-Williamson index; N2P – mean number of alleles across loci (two samples); H2P – mean genic diversity across loci (two sample); V2P – mean allele size variance across loci (two samples); FST – F_{ST}-statistics; LIK – mean index of classification (two samples); DAS – shared allele distance (two samples); DM2 – $(\delta\mu)^2$ distance between two samples; AML – admixture maximum likelihood[11]. The numbers after each statistic refer to the sub-groups: Sub-group 1 – Nambucca Heads, Swansea, and Merimbula; Sub-group 2 – Mallacoota; Sub-group 3 – Ot1 Tasmania; Sub-group 4 – distinct Group Ot2 predominately comprised of individuals from Tasmania (indicated in red in Figs. 1–2).

			***Prob.* (S_{simul}. < $S_{obs.}$)**	
Statistics	Observed	Scenario 6	Scenario 7	Scenario 8
NAL_1_1	15.0000	0.7450	0.8300	0.7840
NAL_1_2	10.5714	0.4165	0.4230	0.4940
NAL_1_3	11.0000	0.3980	0.4465	0.4765
NAL_1_4	10.2857	0.7300	0.9980 (**)	0.9885 (*)
HET_1_1	0.6384	0.0000 (***)	0.0010 (***)	0.0000 (***)
HET_1_2	0.6385	0.0000 (***)	0.0000 (***)	0.0010 (***)
HET_1_3	0.6717	0.0010 (***)	0.0000 (***)	0.0030 (**)
HET_1_4	0.7051	0.0725	0.3870	0.2870
VAR_1_1	5.1877	0.1140	0.1180	0.1070
VAR_1_2	6.5469	0.2330	0.2620	0.2310
VAR_1_3	7.6420	0.3240	0.3470	0.3340
VAR_1_4	8.0216	0.5420	0.5480	0.5110
MGW_1_1	1.8261	1.0000 (***)	1.0000 (***)	1.0000 (***)
MGW_1_2	1.1935	0.9835 (*)	0.9840 (*)	0.9820 (*)
MGW_1_3	1.1324	0.9060	0.9290	0.9370
MGW_1_4	1.0746	0.8930	0.9925 (**)	0.9940 (**)
N2P_1_1&2	16.7143	0.8505	0.8930	0.8710
N2P_1_1&3	17.0000	0.8655	0.8985	0.8790
N2P_1_1&4	16.7143	0.7190	0.9130	0.8965
N2P_1_2&3	13.5714	0.6080	0.6480	0.7035
N2P_1_2&4	14.1429	0.5925	0.8790	0.8840
N2P_1_3&4	14.4286	0.5890	0.8720	0.8710
H2P_1_1&2	0.6419	0.0000 (***)	0.0000 (***)	0.0000 (***)
H2P_1_1&3	0.6503	0.0010 (***)	0.0000 (***)	0.0000 (***)
H2P_1_1&4	0.6898	0.0010 (***)	0.0010 (***)	0.0040 (**)

H2P_1_2&3	0.6577	0.0005 (***)	0.0000 (***)	0.0010 (***)
H2P_1_2&4	0.7269	0.0080 (**)	0.0250 (*)	0.0195 (*)
H2P_1_3&4	0.7290	0.0060 (**)	0.0220 (*)	0.0195 (*)
V2P_1_1&2	5.5302	0.1350	0.1500	0.1280
V2P_1_1&3	5.8945	0.1665	0.1930	0.1600
V2P_1_1&4	6.4166	0.1960	0.2490	0.2140
V2P_1_2&3	7.0868	0.2740	0.3090	0.2800
V2P_1_2&4	7.9790	0.3305	0.4030	0.3720
V2P_1_3&4	8.3774	0.3650	0.4430	0.4170
FST_1_1&2	0.0136	0.7135	0.8205	0.4930
FST_1_1&3	0.0094	0.7060	0.7210	0.3950
FST_1_1&4	0.1364	0.9800 (*)	0.9655 (*)	0.9845 (*)
FST_1_2&3	0.0058	0.4625	0.4860	0.3135
FST_1_2&4	0.1486	0.9760 (*)	0.9705 (*)	0.9750 (*)
FST_1_3&4	0.1126	0.9190	0.8550	0.8830
LIK_1_1&2	1.5444	0.0585	0.0940	0.0505
LIK_1_1&3	1.5562	0.0905	0.1260	0.0685
LIK_1_1&4	2.1124	0.0320 (*)	0.0945	0.0935
LIK_1_2&1	1.4530	0.0530	0.0595	0.0540
LIK_1_2&3	1.4292	0.0450 (*)	0.0420 (*)	0.0500 (*)
LIK_1_2&4	2.1484	0.0515	0.1100	0.1200
LIK_1_3&1	1.5172	0.1085	0.1285	0.0950
LIK_1_3&2	1.4752	0.0555	0.0585	0.0560
LIK_1_3&4	2.0914	0.0355 (*)	0.0840	0.0900
LIK_1_4&1	2.2451	0.3020	0.9145	0.9185
LIK_1_4&2	2.5949	0.5795	0.9820 (*)	0.9615 (*)
LIK_1_4&3	2.4255	0.4470	0.9560 (*)	0.9300
DAS_1_1&2	0.3526	1.0000 (***)	1.0000 (***)	1.0000 (***)
DAS_1_1&3	0.3388	0.9990 (***)	1.0000 (***)	1.0000 (***)
DAS_1_1&4	0.2250	0.9860 (*)	0.9530 (*)	0.9565 (*)
DAS_1_2&3	0.3412	0.9995 (***)	1.0000 (***)	0.9990 (***)
DAS_1_2&4	0.2109	0.9765 (*)	0.9180	0.9325
DAS_1_3&4	0.2243	0.9860 (*)	0.9485	0.9495
DM2_1_1&2	0.0448	0.0170 (*)	0.0090 (**)	0.0130 (*)
DM2_1_1&3	0.2072	0.3865	0.3875	0.2500
DM2_1_1&4	3.5305	0.5620	0.7720	0.8140
DM2_1_2&3	0.0834	0.0615	0.0695	0.0410 (*)
DM2_1_2&4	3.3469	0.5080	0.7480	0.7580
DM2_1_3&4	2.5898	0.3870	0.6490	0.6825
AML_1_3&1&2	0.5010	0.1500	0.1130	0.3285

Table S19. Original parameter estimation and statistics (mean, median, mode and quantiles) of the posterior distribution for three scenarios examined to refine population size changes (bottlenecks) and divergent times for *Octopus tetricus* along the east coast of Australia using the Approximate Bayesian Computation analysis. N1/N2/N3/N4 – effective population size of each cluster; N1b/N2b/N4b – effective number of founder specimens after bottlenecks; t – time in generations; Âµmic – mean mutation rate; snimic – mean Single Nucleotide Insertion/Deletion rate.

Parameter	mean	median	mode	q025	q050	q250	q750	q950	q975
**Scenario 6**									
N1	7.01E+03	7.22E+03	8.00E+03	3.39E+03	4.03E+03	6.00E+03	8.24E+03	9.27E+03	9.49E+03
N2	4.59E+03	4.20E+03	2.41E+03	1.11E+03	1.41E+03	2.71E+03	6.20E+03	9.08E+03	9.53E+03
N3	5.26E+03	5.06E+03	3.95E+03	1.33E+03	1.72E+03	3.30E+03	7.16E+03	9.34E+03	9.66E+03
N4	7.63E+03	7.93E+03	9.54E+03	3.80E+03	4.35E+03	6.43E+03	9.09E+03	9.83E+03	9.92E+03
t0b	1.91E+01	1.53E+01	1.00E+01	1.00E+01	1.00E+01	1.17E+01	2.28E+01	4.24E+01	4.91E+01
N1b	8.41E+03	8.57E+03	9.44E+03	5.89E+03	6.35E+03	7.72E+03	9.29E+03	9.85E+03	9.93E+03
t1	5.25E+01	5.03E+01	4.11E+01	1.71E+01	2.00E+01	3.51E+01	6.82E+01	9.16E+01	9.51E+01
r1	6.51E-01	7.04E-01	8.92E-01	1.73E-01	2.33E-01	5.32E-01	8.11E-01	8.84E-01	8.92E-01
t2	1.22E+02	1.20E+02	1.15E+02	4.54E+01	5.41E+01	8.98E+01	1.55E+02	1.91E+02	1.95E+02
t2b	1.63E+03	1.71E+03	1.91E+03	9.46E+02	1.07E+03	1.48E+03	1.85E+03	1.95E+03	1.97E+03
N4b	2.68E+02	2.86E+02	3.95E+02	6.21E+01	9.06E+01	2.02E+02	3.45E+02	3.90E+02	3.95E+02
t3	1.76E+03	1.82E+03	1.98E+03	1.20E+03	1.32E+03	1.65E+03	1.92E+03	1.99E+03	1.99E+03
Âµmic_1	7.48E-04	7.69E-04	1.00E-03	3.81E-04	4.31E-04	6.30E-04	8.90E-04	9.89E-04	1.00E-03
snimic_1	9.58E-06	1.00E-05	1.00E-05	5.19E-06	6.89E-06	1.00E-05	1.00E-05	1.00E-05	1.00E-05
**Scenario 7**									
N1	5.76E+03	5.79E+03	5.95E+03	2.12E+03	2.60E+03	4.33E+03	7.22E+03	8.80E+03	9.14E+03
N2	6.37E+03	6.40E+03	5.66E+03	2.50E+03	2.97E+03	4.82E+03	8.03E+03	9.53E+03	9.76E+03
N3	6.93E+03	7.08E+03	9.01E+03	2.85E+03	3.44E+03	5.49E+03	8.59E+03	9.73E+03	9.86E+03
N4	7.82E+03	8.42E+03	9.93E+03	2.96E+03	3.71E+03	6.74E+03	9.37E+03	9.90E+03	9.95E+03
t0b	3.86E+01	4.02E+01	5.30E+01	1.24E+01	1.43E+01	2.75E+01	5.08E+01	5.82E+01	5.93E+01
N1b	7.22E+03	7.33E+03	8.34E+03	3.79E+03	4.25E+03	5.96E+03	8.64E+03	9.71E+03	9.84E+03
t1	8.00E+01	8.35E+01	1.00E+02	4.08E+01	4.82E+01	7.12E+01	9.27E+01	9.89E+01	9.95E+01
r1	6.50E-01	7.06E-01	8.82E-01	1.69E-01	2.27E-01	5.25E-01	8.18E-01	8.84E-01	8.93E-01
N4b	3.53E+02	3.68E+02	4.00E+02	2.18E+02	2.52E+02	3.35E+02	3.87E+02	3.98E+02	3.99E+02
t2	1.25E+02	1.26E+02	1.28E+02	5.65E+01	6.61E+01	9.92E+01	1.50E+02	1.84E+02	1.92E+02
t3	2.42E+02	2.32E+02	2.18E+02	1.17E+02	1.33E+02	1.90E+02	2.82E+02	3.76E+02	4.19E+02
Âµmic_1	8.34E-04	8.64E-04	1.00E-03	5.03E-04	5.66E-04	7.50E-04	9.48E-04	1.00E-03	1.00E-03
snimic_1	8.61E-06	1.00E-05	1.00E-05	1.36E-06	2.58E-06	8.30E-06	1.00E-05	1.00E-05	1.00E-05

**Scenario 8**									
N1	6.69E+03	6.90E+03	7.34E+03	2.96E+03	3.55E+03	5.54E+03	8.01E+03	9.18E+03	9.43E+03
N2	5.43E+03	5.26E+03	4.10E+03	1.44E+03	1.75E+03	3.39E+03	7.46E+03	9.47E+03	9.74E+03
N3	5.61E+03	5.48E+03	4.31E+03	1.66E+03	2.03E+03	3.78E+03	7.46E+03	9.48E+03	9.73E+03
N4	6.64E+03	7.05E+03	9.87E+03	1.65E+03	2.19E+03	4.89E+03	8.69E+03	9.77E+03	9.89E+03
t0b	4.27E+01	4.55E+01	6.00E+01	1.49E+01	1.73E+01	3.32E+01	5.40E+01	5.92E+01	5.97E+01
N1b	7.72E+03	7.91E+03	8.53E+03	4.50E+03	5.01E+03	6.70E+03	8.94E+03	9.77E+03	9.89E+03
t1	8.16E+01	8.51E+01	1.00E+02	4.43E+01	5.18E+01	7.36E+01	9.31E+01	9.90E+01	9.96E+01
r1	7.52E-01	8.04E-01	8.91E-01	2.93E-01	4.07E-01	7.04E-01	8.58E-01	8.92E-01	8.96E-01
N4b	3.38E+02	3.52E+02	3.94E+02	2.02E+02	2.27E+02	3.09E+02	3.80E+02	3.96E+02	3.98E+02
t1b	9.89E+01	9.67E+01	9.35E+01	4.20E+01	4.98E+01	7.69E+01	1.20E+02	1.54E+02	1.66E+02
N2b	1.81E+03	1.37E+03	7.40E+02	1.78E+02	2.64E+02	7.38E+02	2.42E+03	4.97E+03	5.88E+03
t2	1.25E+02	1.25E+02	1.15E+02	5.11E+01	6.04E+01	9.67E+01	1.55E+02	1.89E+02	1.94E+02
t3	2.07E+02	1.95E+02	1.82E+02	1.05E+02	1.17E+02	1.60E+02	2.36E+02	3.27E+02	3.84E+02
Âµmic_1	8.05E-04	8.32E-04	1.00E-03	4.64E-04	5.26E-04	7.07E-04	9.29E-04	9.99E-04	1.00E-03
snimic_1	9.40E-06	1.00E-05	1.00E-05	3.94E-06	5.79E-06	9.85E-06	1.00E-05	1.00E-05	1.00E-05

Table S20. Posterior probabilities for the model choice to refine changes in population size (genetic bottlenecks) and divergence times for *Octopus tetricus* along the east coast of Australia using the direct approach of the Approximate Bayesian Computation analysis. * – Type-I error rate; † – Type-II error rate.

	**Scenario 6**	**Scenario 7**	**Scenario 8**
**Posterior Probability**	0.968	0.022	0.010
**95% CI**	[0.8137–1.0000]	[0.0000–0.1506]	[0.0000–0.0972]
**Scenario 6**	78.0%	19.2%†	14.9%†
**Scenario 7**	10.6%*	43.5%	35.1%
**Scenario 8**	11.4%*	37.3%	50.0%

References

1. Jombart, T., Devillard, S. & Balloux, F. Discriminant analysis of principal components: a new method for the analysis of genetically structured populations. *BMC Genet* **11,** 94; 10.1186/1471-2156-11-94 (2010).
2. Earl, D. A. & vonHoldt, B. M. STRUCTURE HARVESTER: a website and program for visualizing STRUCTURE output and implementing the Evanno method. *Conserv. Genet. Resour.* **4,** 359–361; 10.1007/s12686-011-9548-7 (2012).
3. Folmer, O., Black, M., Hoeh, W., Lutz, R. & Vrijenhoek, R. DNA primers for amplification of mitochondrial cytochrome c oxidase subunit from diverse metazoan invertebrates. *Mol. Mar. Biol. Biotechnol.* **3,** 294–299 (1994).
4. Guzik, M. T., Norman, M. D. & Crozier, R. H. Molecular phylogeny of the benthic shallow-water octopuses (Cephalopoda:Octopodinae). *Mol. Phylogenet. Evol.* **37,** 235–248; 10.1016/j.ympev.2005.05.009 (2005).
5. Amor, M. D., Norman, M. D., Cameron, H. E. & Strugnell, J. M. Allopatric Speciation within a Cryptic Species Complex of Australasian Octopuses. *PLoS ONE* **9,** e98982; 10.1371/journal.pone.0098982 (2014).
6. Posada, D. jModelTest: Phylogenetic model averaging. *Mol. Biol. Evol.* **25,** 1253–1256; 10.1093/molbev/msn083 (2008).
7. Akaike, H. A new look at the statistical model identification. *IEEE Trans. Automat. Contr.* **19,** 716–723 (1974).
8. Guindon, S. *et al*. New algorithms and methods to estimate Maximum-Likelihood phylogenies: Assessing the performance of PhyML 3.0. *Syst. Biol.* **59,** 307–321; 10.1093/sysbio/syq010 (2010).
9. Ronquist, F. & Huelsenbeck, J. P. MrBayes 3: Bayesian phylogenetic inference under mixed models. *Bioinformatics* **19,** 1572–1574; 10.1093/bioinformatics/btg180 (2003).
10. Rambaut, A., Drumond, A. J. & Suchard, M. Tracer. MCMC Trace Analysis Tool version 1.6.0, 2003–2013. http://www.molecularevolution.org/software/phylogenetics/tracer (2013).
11. Choisy, M. P., Franck, P. & Cornuet, J. M. Estimating admixture proportions with microsatellites: comparison of methods based on simulated data. *Mol. Ecol.* **13,** 955–968; 10.1111/j.1365-294X.2004.02107.x (2004).
